# Enzyme- and DNAzyme-Driven Transient Assembly of DNA-Based Phase-Separated Coacervate Microdroplets

**DOI:** 10.1021/jacs.5c00637

**Published:** 2025-04-30

**Authors:** Yunlong Qin, Yang Sung Sohn, Rachel Nechushtai, Fan Xia, Fujian Huang, Itamar Willner

**Affiliations:** † The Institute of Chemistry, The Hebrew University of Jerusalem, Jerusalem 91904, Israel; ‡ The Institute of Life Science, The Hebrew University of Jerusalem, Jerusalem 91904, Israel; § State Key Laboratory of Geomicrobiology and Environmental Changes, Faculty of Materials Science and Chemistry, China University of Geosciences, Wuhan 430074, China

## Abstract

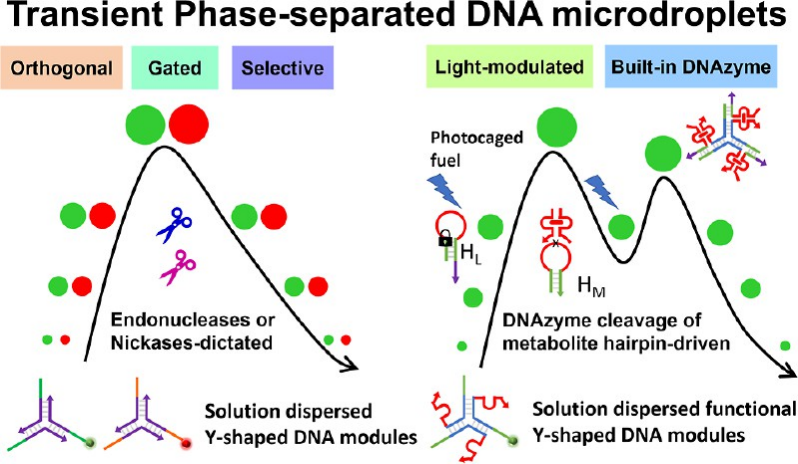

An assembly of dissipative, transient, DNA-based microdroplet (MD) coacervates in the presence of auxiliary enzymes (endonucleases and nickases) or MD-embedded DNAzyme is introduced. Two pairs of different Y-shaped DNA core frameworks modified with toehold tethers are cross-linked by complementary toehold-functionalized duplexes, engineered to be cleaved by EcoRI or HindIII endonucleases, or cross-linked by palindromic strands that include pre-engineered Nt.BbvCI or Nb.BtsI nicking sites, demonstrating transient evolution/depletion of phase-separated MD coacervates. By mixing the pairs of endonuclease- or nickase-responsive MDs, programmed or gated transient formation/depletion of MD frameworks is presented. In addition, by cross-linking a pre-engineered Y-shaped core framework with a sequence-designed fuel strand, phase separation of MD coacervates with embedded Mg^2+^-DNAzyme units is introduced. The DNAzyme-catalyzed cleavage of a ribonucleobase-modified hairpin substrate, generating the waste product of the metabolite fragments, leads to the metabolite-driven separation of the cross-linked coacervates, resulting in the temporal evolution and depletion of the DNAzyme-functionalized MDs. By employing a light-responsive caged hairpin structure, the light-modulated fueled evolution and depletion of the DNAzyme-active MDs are presented. The enzyme- or DNAzyme-catalyzed transient evolution/depletion of the MD coacervates provides protocell frameworks mimicking dynamic transient processes of native cells. The possible application of MDs as functional carriers for the temporal, dose-controlled release of loads is addressed.

## Introduction

Membraneless condensates loaded with proteins or oligonucleotides play important roles in regulating cellular functions, such as RNA metabolism or signal transduction.^1−6^ Many of these processes demonstrate spatiotemporal, switchable separation and condensation, accompanied by size changes and controlled biochemical functionalities.^7−9^ Substantial research efforts are directed to develop synthetic coacervates emulating the functions of native membraneless organelles.^10−12^ These included the complexation of oppositely charged polymers^13,14^ or dipole-functionalized surfactants.^15^ Moreover, physically or chemically triggerable constituents were integrated in the coacervates to stimulate dynamic shapes and functions in the membraneless frameworks. For example, by integrating a phosphate kinase in RNA–peptide coacervates, the shapes of phase-separated frameworks controlled by the degree of peptide phosphorylation were demonstrated.^16^ Similarly, GTP-driven polymerization of the filamenting temperature-sensitive mutant Z (FtsZ) protein in coacervates led to dynamic deformation and fission of the synthetic organelles.^17^ In addition, incorporation of different enzymes in the mixtures of coacervates led to intercommunication of the droplets and the activation of programmable biocatalytic cascades.^18,19^ Nevertheless, to further develop biomimetic membraneless condensates, the assembly of synthetic organelles of enhanced structural and functional complexities revealing gated, cascaded, or orthogonal programmable transport and catalytic and morphological dynamic features is essential. Particularly, out-of-equilibrium transient formation, dissociation, and fission of functional coacervates, following the concepts of recently reported fueled formation and dissociation of oligonucleotide-based polymer fibers,^20,21^ could be challenging.

The base sequence encoded in oligonucleotides (DNA) provides substantial structural and functional information that can be utilized for the assembly of membraneless synthetic condensates.^22^ The dictated hybridization of duplex oligonucleotides and their displacement by fuel strands,^23−25^ the triggered reversible reconfiguration of the DNA strand by auxiliary stimuli signals, such as pH,^26,27^ light,^28−30^ formation/dissociation of G-quadruplex^31,32^ or metal-ion bridges,^33,34^ and the selective enzyme-guided cleavage of duplex oligonucleotides by endonucleases, and nickases,^35−37^ or ligation of single strands by ligases,^38^ provide a rich “tool-box” to temporally reconfigure DNA structures. Indeed, previous studies have implemented stimuli-responsive supramolecular oligonucleotide structures to assemble DNA switches^39^ and machines,^40−42^ to stimulate reversible DNA structures,^43,44^ and to develop stimuli-responsive materials, such as stiffness-switchable hydrogels.^45−47^ These systems and materials were broadly applied to develop programmable catalytic systems,^48^ stimuli-responsive drug delivery^49−51^ and imaging carriers,^52^ sensors,^53,54^ and optical switching devices.^55−57^ In addition, the programmable recognition and dynamic switchable reconfiguration properties of oligonucleotides were applied to assemble programmable phase-separated microdroplets (MDs)^58^ or DNA-based condensates as membraneless organelle models.^59^ For example, cross-linking of toehold-modified three-arm- or four-arm-shaped oligonucleotide structures,^60,61^ or enzyme-responsive star-like units,^61,62^ resulted in the formation and dynamic fission of DNA MDs. In addition, interhybridization of repeat units associated with entangled rolling circle amplification (RCA)-generated products,^63^ condensation of palindromic domains in DNA single strands, and kinetic trapping of polyadenine-rich single strands^64^ were applied to generate phase-separated coacervates. Also, dynamic microcompartmentalization of DNA condensates by complementary strands exhibiting different hybridization lengths applying the reaction-diffusion mechanistic pathway was realized.^59^

While substantial progress in the synthesis of DNA-based phase-separated microdroplet coacervates and condensates was accomplished, temporal transient operation of DNA coacervates and, particularly, dynamic, transient, microdroplet-guided systems is scarce, despite the rapid advances in developing dissipative DNA reaction modules.^65−67^ Light-stimulated switchable formation and separation of phase-separated DNA MDs in the presence of a positively charged trans/cis azobenzene intercalator were demonstrated.^8^ Photoisomerization of *trans*-azobenzene/duplex DNA-stabilized MDs led to the *cis*-state-stimulated separation of the condensates, and light reisomerization of the *cis*-azobenzene units to the *trans*-state resulted in the recovery of the MDs. In addition, pH-responsive DNA coacervates undergoing light-stimulated cyclic transient and oscillatory formation and dissociation in the presence of a photoacid were demonstrated.^68^ Also, the ATP-fueled ligation of monovalent complementary toehold duplexes led to the multivalent interhybridization of the complementary strands, yielding phase-separated DNA coacervates. By encoding endonuclease-specific domains in the multivalent cross-linking units, transient dissolution of the coacervates was demonstrated.^18^ By the integration of glucose oxidase (GOx) and horseradish peroxidase (HRP) in the multivalent coacervates, the transient GOx/HRP biocatalytic cascade was driven by the transient confined phase-separated DNA coacervates.

In the present study, we introduce innovative concepts that advance the topic of dissipative transient phase-separated MDs by conjugating catalytic functions to the dynamic morphologies of phase-separated coacervates. Specifically, the study aims to demonstrate the ability to control the growth and temporal depletion of coacervates by auxiliary catalytic agents (enzymes) or microdroplet-embedded catalytic units (DNAzymes). This will not only demonstrate the capability to control the “fate” of a protocell but also enable the temporal controlled release of loads from the protocell frameworks. We report on an alternative approach to assemble transient phase-separated MDs by the cross-linking of Y-shaped DNA units with programmable enzyme-responsive cross-linking units (enzyme = endonucleases or nickases). The concomitant biocatalyzed cleavage of the cross-linking domains leads to the transient formation and depletion of the phase-separated condensates. By mixing DNA coacervates responding to different endonucleases, gated phase separation of MDs or selective transient depletion of coacervates is introduced. In addition, we demonstrate the integration of active split Mg^2+^-ion-dependent 10−23 DNAzyme units^69^ into the coacervates yielding a functional framework that in the presence of an auxiliary hairpin substrate leads to the metabolite-driven transient depletion of the cross-linked DNA MDs. Moreover, by coupling a photocaged fuel strand together with a hairpin substrate to the Y-shaped DNA core framework, the light-triggered transient formation/depletion of the coacervate microdroplet and the light-modulated shapes (sizes) of the condensates are demonstrated. The method and the diverse stimuli-triggered reconfiguration means of DNA strands pave the way to assemble many other transient phase-separated MDs using pH, triplex, G-quadruplexes, miRNAs, aptamer/ligand, or light as guiding triggers.

## Results and Discussion

Enzymes, such as nickase^70^ or endonucleases,^71^ as well as DNAzymes,^72^ were previously used to control transient DNA circuitries. These reaction modules and circuitries were used to control transient biocatalytic cascades,^73^ to operate dissipative constitutional dynamic networks,^74^ to control artificial photosynthetic systems,^75^ and to guide the transient operation of transcription machineries, and they are used for the transient operation of DNAzyme-catalyzed or temporal activation/inhibition of thrombin^76^ as the blood-clotting agent. These biocatalysts are now used to perform the transient operation of assembling DNA-based phase-separated coacervate MDs.

**Figure 1:**
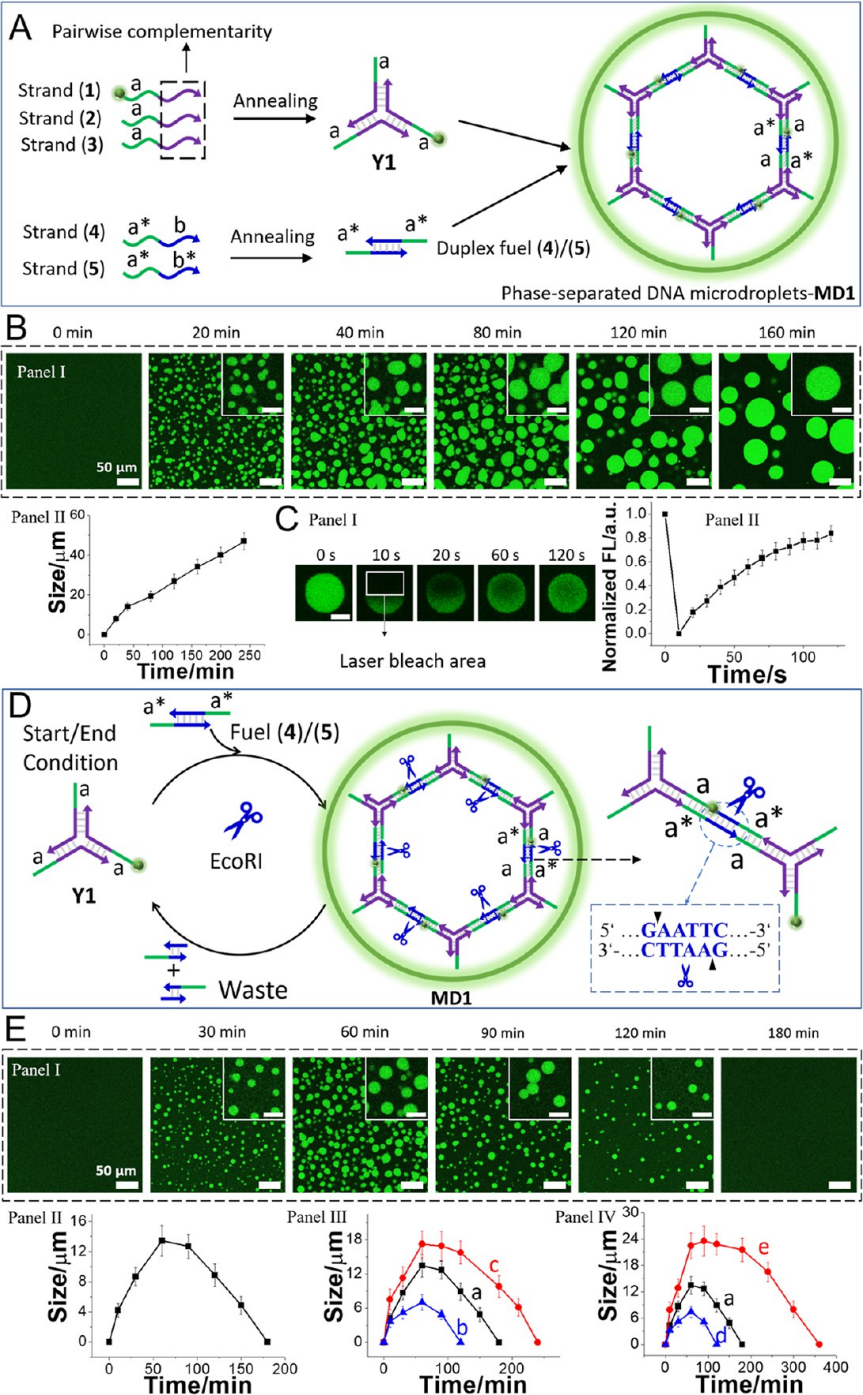
(A) Schematic assembly of a phase-separated microdroplet (MD) condensate **MD1** by the cross-linking of toehold-modified Y-shaped DNA modules **Y1** with complementary toehold-functionalized duplex units. (B) Panel I—Confocal fluorescence microscopy images corresponding to the growth of the duplex-bridged Y-shaped modules; scale bar = 50 μm, inset scale bar = 20 μm. Panel II—Average temporal diameter changes of the phase-separated **MD1** condensates. (Average diameters derived by analyzing four different imaged frames. Error bars derived from 3 different experiments.) (C) Panel I—Confocal fluorescence microscopy images (scale bar = 5 μm) corresponding to the time-dependent recovery of a laser-induced bleached domain in the MD. Panel II—Temporal fluorescence intensity changes of the bleached domain. (D) Schematic assembly of a dissipative, endonuclease (EcoRI)-guided transient evolution and depletion of phase-separated MDs formed by the cross-linking of a Y-shaped framework **Y1** with a pre-engineered EcoRI-endonuclease-cleavable duplex. (E) Panel I—Temporal confocal fluorescence microscopy images (scale bar = 50 μm, inset scale bar = 20 μm). Panel II—Temporal size (diameter) changes corresponding to the transiently evolved and depleted EcoRI-responsive MDs, in the presence of **Y1**, 10 μM, EcoRI, 8 U/μL, and (**4**)/(**5**), 15 μM. Panel III—Transient size changes of MDs generated in the presence of **Y1**, 10 μM, EcoRI, 8 U/μL, and different concentrations of the fuel bridging duplex: (a) 15 μM, (b) 12 μM, and (c) 18 μM. Panel IV—Transient size changes of MDs generated in the presence of a Y-shaped module, 10 μM, fuel bridging duplex, 15 μM, and different concentrations of EcoRI: (a) 8 U/μL, (d) 12 U/μL, and (e) 4 U/μL.

Figure 1A depicts the method to assemble the endonuclease-responsive phase-separated microdroplets, MDs, **MD1** exhibiting transient dissipative features. Y-shaped DNA modules **Y1**, generated by the annealing of three pre-engineered single strands (**1**), (**2**), and (**3**) that include toehold tethers “a” in each arm, were challenged with the fuel duplex (**4**)/(**5**) that includes in each of the strands a toehold sequence “a*” complementary to the toehold tethers associated with the **Y1** frameworks. The process leads to 3D cross-linking of the **Y1** modules and to the phase-separated formation of **MD1**. As 10% of the strand (**1**) is functionalized with the green fluorescent dye, fluorescein, the formation of **MD1** is followed by fluorescence confocal microscopy (Figure 1B). Within a time-interval of ca. 160 min, the dynamic growth of the MDs proceeds, yielding green fluorescent droplets exhibiting an average size distribution of 30 μm. The MDs reveal a fluidic, liquid-like behavior, rather than a gel-like system, reflected by the rapid healing recovery of the fluorescence of MDs subjected to laser confocal microscopy-bleached domain (Figure 1C). The bleached domain is recovered within ca. 2 min, supporting a liquid-like, diffusive DNA constituent in the MD microenvironments.^58^ (For further discussion addressing the fluidic nature of the droplets, see page S9, Supporting Information.) The fuel duplexes (**4**)/(**5**) were, however, engineered to be cleaved by the endonuclease EcoRI. Accordingly, subjecting the Y-shaped module **Y1** to the cross-linking, fuel duplexes (**4**)/(**5**), in the presence of EcoRI, results in the controlled growth and competitive depletion of MDs, as schematically depicted in Figure 1D. The cross-linking of the Y-shaped module **Y1** by (**4**)/(**5**) leads to the formation of the cross-linked **MD1**, yet the concomitant EcoRI cleavage of the bridging (**4**)/(**5**) cross-linking units inhibits the growth of the droplets and ultimately induces the transient dissipative depletion of **MD1** (the assembly/depletion process of the **MD1** framework was also supported by gel electrophoretic experiment, see Figure S1 and the accompanying discussion). Figure 1E, Panels I and II, depict the transient time-dependent formation and depletion of **MD1** in the presence of **Y1** (10 μM), EcoRI (8 U/μL), and (**4**)/(**5**) (15 μM). Within ca. 60 min, the growth of the MDs to an average maximum size of 12 μm proceeds, and afterward, the temporal size and content of the MDs decrease, leading to the complete depletion of MDs after ca. 180 min. As expected, the sizes of the MDs and their dynamic transient depletion rates are controlled by the concentration of the fuel duplexes (**4**)/(**5**) and the concentration of the regulating depleting endonuclease. As the concentration of the fuel duplexes (**4**)/(**5**) is higher, larger MDs are formed, and the catalyzed depletion is prolonged (Figure 1E, Panel III). In addition, increasing the concentration of the endonuclease decreases the average peak sizes of the MDs and enhances their transient depletion (Figure 1E, Panel IV).

A similar approach was applied to organize a second type of HindIII-driven red-fluorescent-labeled dissipative MDs, **MD2**. In this system, a second reaction module **Y2** composed of strands (**6**), (**7**), and (**8**), which included single-stranded toehold domains “c” and labeled with the red fluorescent label Cy5, was cross-linked with the “c*” toehold-modified duplexes (**9**)/(**10**) to yield the phase-separated red fluorescent **MD2**, Figure S2. The duplexes (**9**)/(**10**) were engineered, however, to be cleaved by the endonuclease HindIII, leading to the transient formation and dissipative depletion of the MDs **MD2**, as shown in Figure S3. For the gel electrophoretic characterization of the Hind III-responsive framework of **MD2**, see Figure S4, Supporting Information. It should be, however, noted that the kinetics of the duplex-fueled evolution of the EcoRI- or HindIII-responsive MDs is affected by the thermodynamic stabilities of the fuel strand complementarities with the respective Y-shaped module assembling the coacervate MDs. These features affect the rates and sizes of the formed MDs (see Figure S5 and the accompanying discussion).

**Figure 2:**
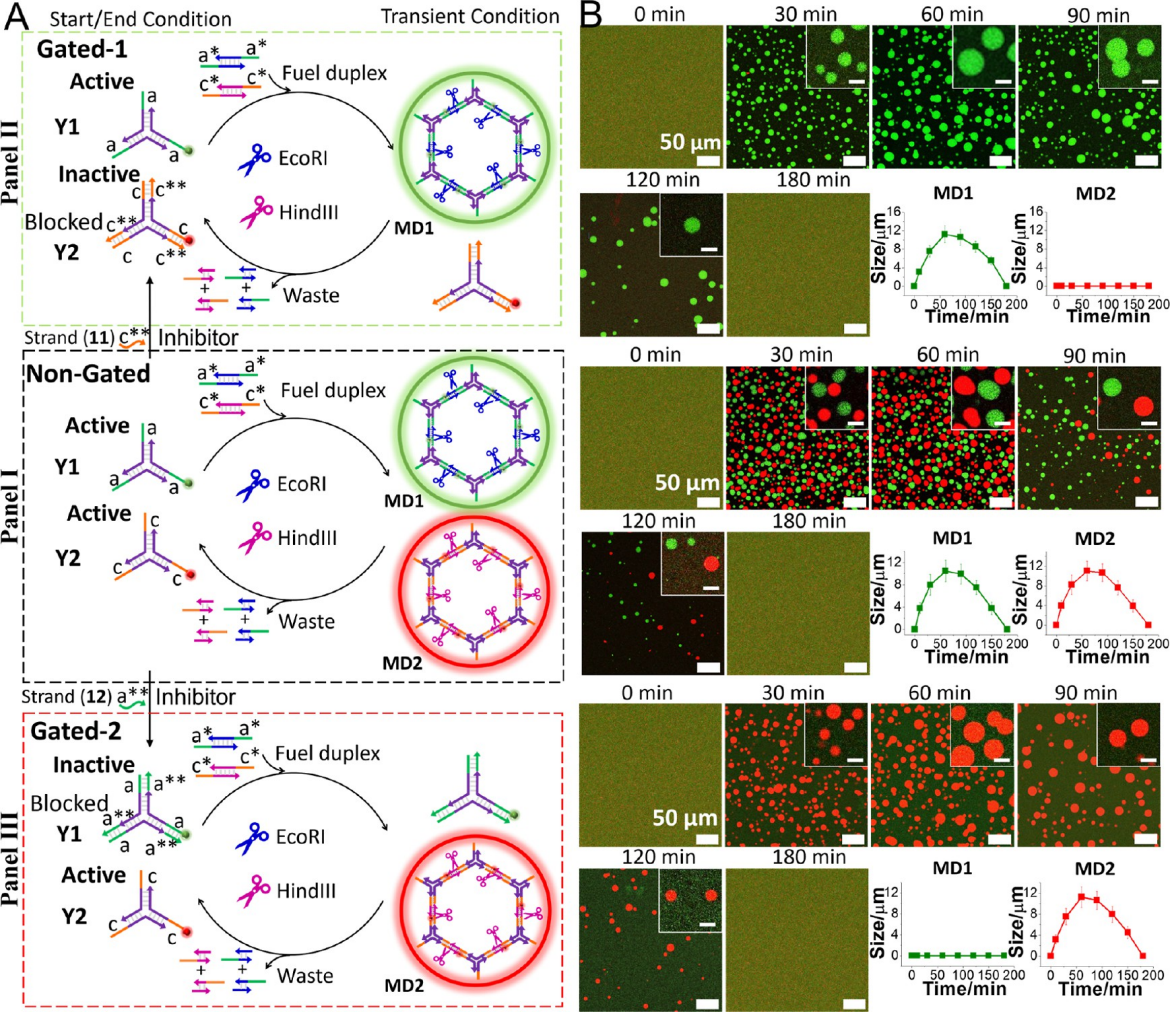
(A) Inhibitor-guided gated transient formation/depletion of target endonuclease-responsive MDs within a mixture of EcoRI-/HindIII-responsive MDs (**MD1** and **MD2**) and (B) corresponding temporal confocal fluorescence microscopy images and accompanying transient size changes of the gated MDs (scale bar = 50 μm, inset scale bar = 10 μm). Panel I—Non-gated MD assembly operating the dual formation and depletion of the transient EcoRI- and HindIII-responsive MDs (**MD1** and **MD2**). Panel II—Inhibitor (**11**)-gated blockage of HindIII-responsive MDs (**MD2**) and selective operation of transient evolution/depletion of EcoRI-responsive MDs (**MD1**). Panel III—Inhibitor (**12**)-gated blockage of EcoRI-responsive MDs (**MD1**) and selective operation of transient evolution/depletion of HindIII-responsive MDs (**MD2**). The concentrations of EcoRI and HindIII are 8 U/μL.

The mixture of EcoRI- and HindIII-responsive MDs was then applied to develop a gated, transient, dissipative depletion of the target MDs, in the presence of appropriate inhibitors (Figure 2). Subjecting the mixture of “active” **Y1** and **Y2** green/red modules to the fuel duplexes (**4**)/(**5**) and (**9**)/(**10**), in the presence of the endonucleases EcoRI and HindIII, resulted in the transient, concomitant formation and depletion of the green fluorescent and red fluorescent MDs, **MD1** and **MD2**, (non-gated) (Figure 2A, Panel I). Subjecting, however, the mixture **Y1** and **Y2** to the inhibitor strand (**11**) “c**” results in the selective hybridization-induced inhibition of the toehold arms associated with the framework **Y2** (“inactive”). As a result, treatment of the mixture **Y1** (“active”) and **Y2** (“inactive”) with the fuel strands (**4**)/(**5**) and (**9**)/(**10**) results in the gated transient formation and depletion of frameworks **MD1**, while the formation of the **Y2**-based MDs, **MD2**, is fully blocked (gated 1) (Figure 2A, Panel II). (Note that the duplexes (**9**)/(**10**) cannot displace the inhibitor, since the inhibitor “c**” forming the duplex “c/c**” is designed to yield a duplex of enhanced stability, as compared to “c/c*”.) Similarly, blocking of the **Y1** toehold units with the inhibitor strand “a**” results in the gated operation of the (**9**)/(**10**)-guided, cross-linking of the red fluorescent coacervate **MD2**, undergoing transient formation and depletion in the presence of EcoRI and HindIII (Figure 2A, Panel III). The temporal confocal fluorescence microscopy images and temporal size changes of the non-gated transient formation/depletion of green fluorescent **MD1** and red fluorescent **MD2** are displayed in Figure 2B, Panel I. Obviously, subjecting the mixture of fuel (**4**)/(**5**) and (**9**)/(**10**) to non-gated “active” **Y1** module and “active” **Y2** module, in the presence of EcoRI and HindIII, led to the growth of a mixture of orthogonal separated MDs, **MD1** (green) and **MD2** (red), with similar sizes of ca. 11 μm within a time interval of 60 min. The concomitant EcoRI and HindIII degradation of the MDs resulted in the transient depletion of the two MDs and full depletion after a time interval of 180 min. The temporal confocal fluorescence microscopy images and temporal size changes of the inhibitor (**11**)-gated (gated 1) transient formation/depletion of green fluorescent **MD1** are displayed in Figure 2B, Panel II. While the assembly of the green fluorescent **MD1** within a time interval of 60 min is observed (MD sizes ca. 12 μm), followed by the dissipative depletion of MDs within a time interval of 180 min, the red fluorescent **MD2** was totally blocked and not observed during the time interval because of the inhibitor (**11**)-induced inactivation of **Y2** modules. Alternatively, the temporal confocal fluorescence microscopy images and temporal size changes of the inhibitor (**12**)-gated (gated 2) transient formation/depletion of red fluorescent **MD2** are displayed in Figure 2B, Panel III. While the temporal formation of the red fluorescent **MD2** is observed (sized ca. 12 μm within a time interval of 60 min) accompanied by the depletion of **MD2** within a time interval of 180 min, the green fluorescent **MD1** is not observed during the time interval, due to the inhibitor (**12**)-induced inactivation of the **Y1** module. Moreover, the gated operation of the two MDs, **MD1** and **MD2**, can be tuned by the concentrations of the respective endonucleases. The results are presented in Figures S6–S8 and the accompanying discussion. (Specificity of phase-separated **MD1** and **MD2** and the orthogonal assembly of mixed green fluorescent **MD1** and red fluorescent **MD2** are discussed in Figures S9 and S10.)

**Figure 3:**
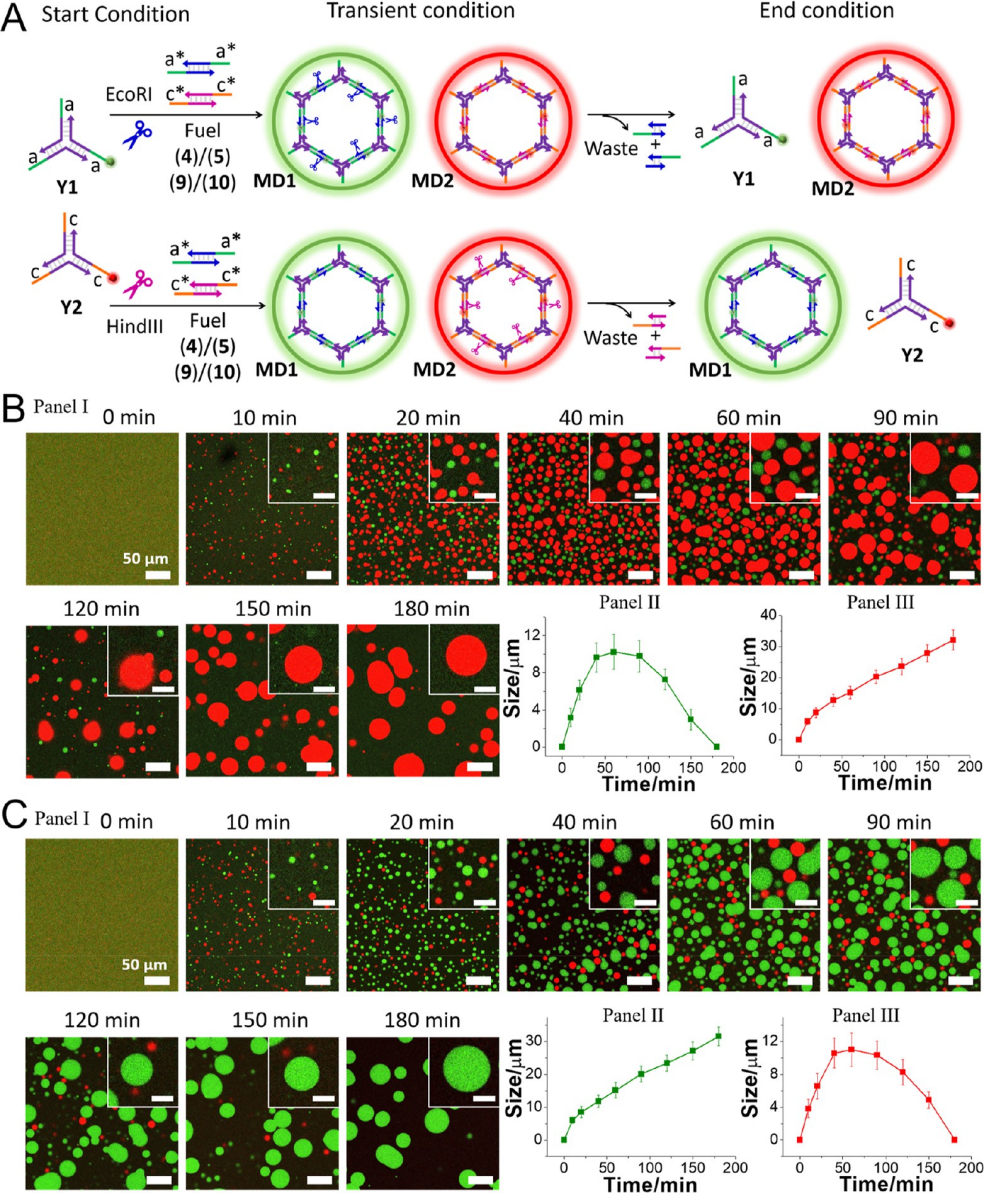
(A) Schematic selective transient assembly/depletion of phase-separated MDs, **MD1** and **MD2**, using a mixture of Y-shaped modules **Y1** and **Y2** and a mixture of fuel (**4**)/(**5**) and (**9**)/(**10**), in the presence of a single endonuclease, EcoRI or HindIII. (B) Temporal confocal fluorescence microscopy images (scale bar = 50 μm, inset scale bar = 20 μm) (Panel I) and temporal average size changes of **MD1** (Panel II) and **MD2** (Panel III), corresponding to the selective assembly/depletion of phase-separated MDs, **MD1** and **MD2**, using a mixture of Y-shaped modules **Y1** and **Y2**, and a mixture of fuel (**4**)/(**5**) and (**9**)/(**10**), in the presence of a single endonuclease, EcoRI. (C) Temporal confocal fluorescence microscopy images (scale bar = 50 μm, inset scale bar = 20 μm) (Panel I) and temporal average size changes of **MD1** (Panel II) and **MD2** (Panel III), corresponding to selective assembly/depletion of phase-separated MDs, **MD1** and **MD2**, using a mixture of Y-shaped modules **Y1** and **Y2**, and a mixture of fuel (**4**)/(**5**) and (**9**)/(**10**), in the presence of a single endonuclease, HindIII.

The selective transient formation/depletion of **MD1** or **MD2** in the mixture of MDs is schematically presented in Figure 3A. Subjecting the mixture of **Y1** and **Y2** core frameworks to the fuels (**4**)/(**5**) and (**9**)/(**10**), in the presence of only EcoRI, leads to the selective transient evolution/depletion of **MD1**, where **MD2** is only formed, without being depleted (Figure 3B, Panels I, II, and III). Similarly, treatment of the mixture of **Y1** and **Y2** modules with the fuels (**4**)/(**5**) and (**9**)/(**10**), in the presence of only HindIII, leads to the selective transient evolution and depletion of **MD2**, while **MD1** is evolved without further degradation (Figure 3C, Panels I, II, and III).

**Figure 4:**
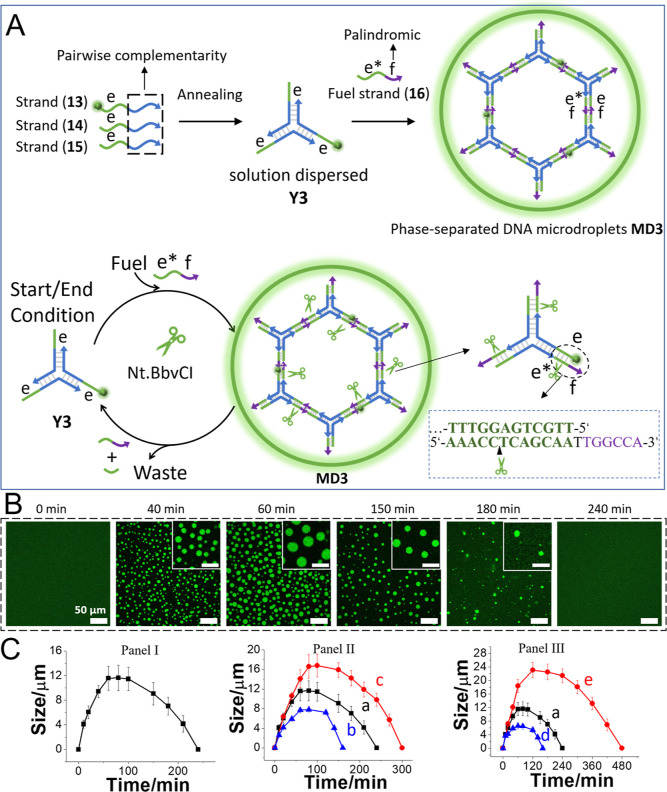
(A) Schematic assembly of nickase-controlled phase-separated transient evolution and depletion of MDs through the fueled cross-linking of Y-shaped DNA frameworks **Y3** by a palindromic fuel strand (**16**). (B) Temporal confocal fluorescence microscopy images corresponding to nickase-controlled palindromic strand-fueled evolution/depletion of the MDs (scale bar = 50 μm, inset scale bar = 20 μm). (C) Panel I—Temporal size changes of the transiently evolved/depleted MDs in the presence of Y3, 10 μM, fuel, 30 μM, and nickase (Nt.BbvCI), 0.8 U/μL. Panel II—Temporal size changes of the nickase-controlled MDs in the presence of **Y3**, 10 μM, Nt.BbvCI, 0.8 U/μL, and different concentrations of the palindromic fuel (**16**): (a) 30 μM, (b) 24 μM, and (c) 36 μM. Panel III—Temporal size changes of the nickase-controlled MDs in the presence of Y3, 10 μM, fuel (**16**), 30 μM, and different concentrations of Nt.BbvCI: (a) 0.8 U/μL, (d) 1.2 U/μL, and (e) 0.4 U/μL. (Average diameters derived by analyzing 4 different imaged frames. Error bars derived from 3 different experiments.)

A similar concept demonstrating the transient biocatalyzed assembly and depletion of the phase-separated MDs was achieved using nickase (Nt.BbvCI) as the catalytic agent stimulating the competitive temporal depletion of the MDs (Figure 4A). A Y-shaped framework **Y3** composed of strands (**13**), (**14**), and (**15**) was cross-linked by the palindromic strand (**16**), yielding the cross-linked phase-separated framework **MD3** (where 10% of the strand (**13**) is doped with the green fluorescent probe fluorescein). The confocal fluorescence microscopy images corresponding to the assembly of the green fluorescent phase-separated MDs, **MD3**, and the liquid features of the resulting **MD3** (photobleaching experiments) are presented in Figure S11 and the accompanying discussion. The cross-linking strand (**16**) was engineered, however, to include the sequence-specific domain to be cleaved by nickase (Nt.BbvCI). Accordingly, the fuel-triggered phase-separated formation of **MD3** is accompanied by the competitive nickase-induced cleavage of the cross-linking bridging units, leading to the temporal transient depletion of **MD3** (gel electrophoretic analysis of the assembly/depletion process is provided in Figure S12). Figure 4B depicts the confocal fluorescence microscopy images of **MD3** at different times of the dynamic evolution and transient biocatalyzed depletion. The MDs reveal a time-dependent temporal size increase for ca. 60 min, followed by a transient temporal size and content decrease of the MDs that are fully depleted after ca. 240 min. Figure 4C, Panel I depicts the time-dependent size changes of the MDs upon the dynamic evolution and concomitant biocatalyzed depletion of the MDs. The transient peak sizes of the MDs and their depletion rates are controlled by the concentrations of the cross-linking fuel strands, Panel II, and the concentrations of nickase, Panel III. As the fuel strand concentration increases, the peak sizes of the MDs are higher, and as the concentration of nickase increases, the depletion rates are enhanced and the peak sizes of the evolved MDs decrease.

Furthermore, a second nickase (Nb.BtsI)-dictated transient assembly and depletion of red fluorescent phase-separated MDs, **MD4**, are presented in Figures S13–S15 and the accompanying discussion. The specificity of phase-separated MDs, **MD3**, or **MD4** in a foreign nickase is introduced in Figure S16. Moreover, the orthogonal and selective transient formation/depletion of two kinds of mixed phase-separated MDs, **MD3** (green fluorescence) and **MD4** (red fluorescence), was realized, and the results are presented in Figures S17–S19 and the accompanying discussion. It is important to note that the reported endonuclease- and nickase-responsive MD systems demonstrated comparable evolution rates and coacervate dimensions. Nevertheless, the results revealed that to reach the respective MDs, different concentrations of the enzymes and tunable control over the fuel sequences bridging the Y-shaped elements need to be appropriately adjusted. Furthermore, the fuel-triggered formation of the different intermediate MDs is associated with appropriate energy inputs, and the enzymatic depletion of the MDs involves an energy release. The balance of energy input/output values for the different dissipative systems is provided in Figures S20–S24. For the estimation of the catalytic rates of the different enzymes driving the transient operation of the condensates, see Supporting Information, page S49.

**Figure 5:**
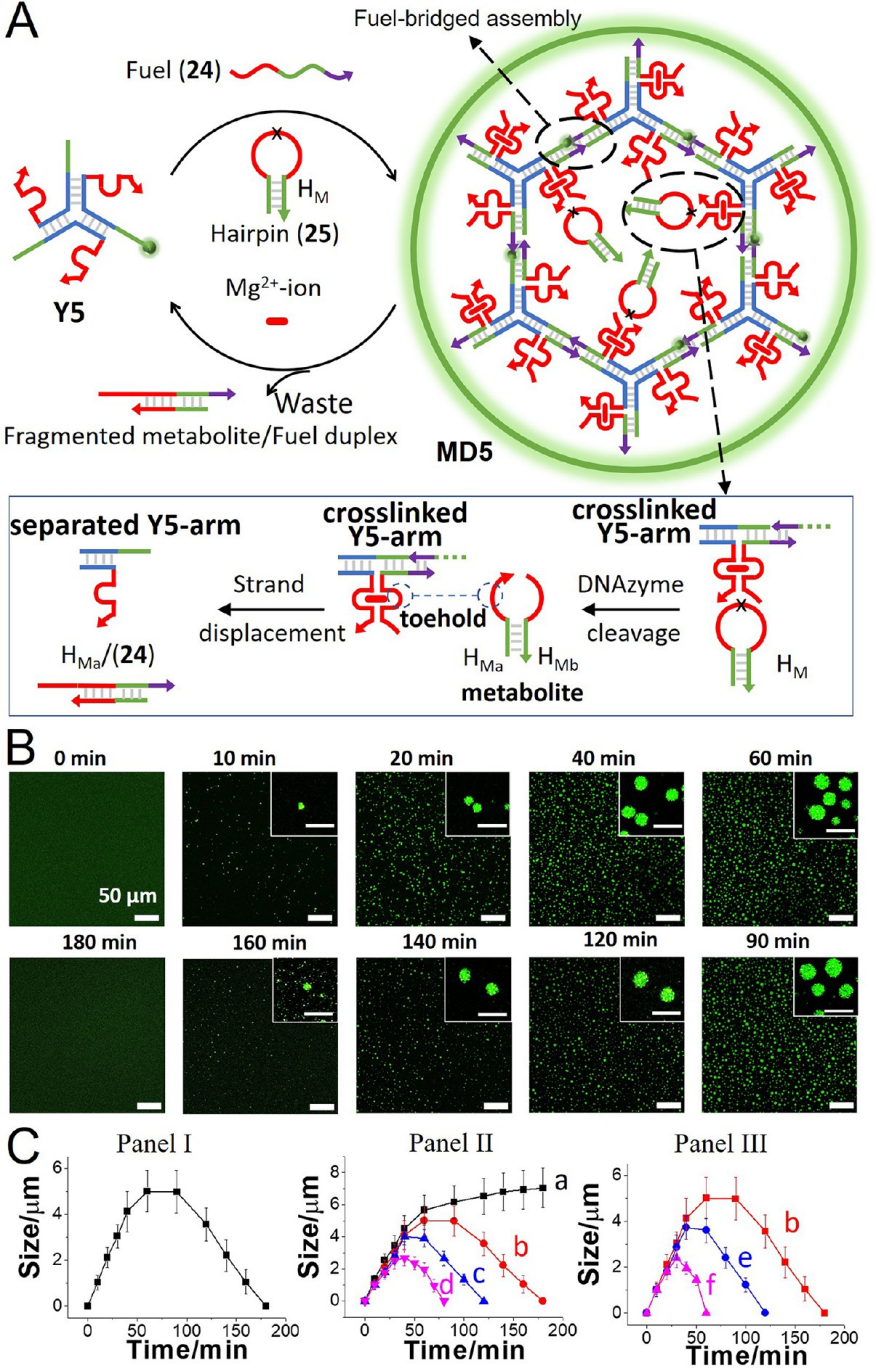
(A) Schematic fuel-driven assembly of Mg^2+^-ion-dependent DNAzyme cross-linked MD coacervates **MD5** undergoing temporal metabolite depletion in the presence of a ribonucleobase-modified hairpin acting as a substrate of the DNAzyme. (B) Temporal confocal fluorescence microscopy images (scale bar = 50 μm, inset scale bar = 10 μm) corresponding to fuel (**24**)-triggered (24 μM) transient evolution/depletion of **MD5**, in the presence of **Y5** (8 μM) and H_M_ (18 μM). (C) Panel I—Temporal size changes of the MDs corresponding to the transient evolution and DNAzyme-catalyzed depletion of the MDs by subjecting fuel (**24**) (24 μM) to a mixture of **Y5** (8 μM) and H_M_ (18 μM). (C) Panel II—Curve (a): Temporal size changes of the MDs in a control system consisting of the (**24**)-fueled (24 μM) cross-linking of the Y-shaped module **Y5** (8 μM) in the presence of a mutated hairpin (**26**) (24 μM), lacking the DNAzyme-cleavable site in the loop domain. Curves (b–d): Temporal transient MD sizes associated with the evolution/depletion of the MDs in the presence of different fuel cross-linker concentrations: (b) 24 μM, (c), 21 μM, and (d) 18 μM. In all experiments, the concentration of **Y5** was 8 μM and that of H_M_ was 18 μM. Panel III—Temporal transient size changes of the MDs evolved in the presence of different H_M_ concentrations: (b) 18 μM, (e) 21 μM, and (f) 24 μM. In all experiments, **Y5**, 8 μM, fuel (**24**), 24 μM (error bars in all experiments are derived from *N* = 3 experiments, analyzing in each experiment 4 imaged frames).

The results presented so far employed auxiliary biocatalysts as functional constituents driving the transient, dissipative behavior of the coacervate DNA MDs. To approach cell-like functionalities into the DNA MDs, integration of the catalytic units into the MD framework driving the dynamic transient properties of the DNA condensates is desirable. Toward this goal, we integrated a catalytic DNAzyme unit into the DNA MD framework. Figures 5A and S25 depict the assembly of DNAzyme-functionalized MDs, leading to the fuel-driven transient formation and depletion of DNA MDs, **MD5**. Annealing of three strands (**21**), (**22**), and (**23**) leads to the assembly of the Y-shaped module **Y5**. The strands are pre-engineered to include in each arm the Mg^2+^-ion-dependent DNAzyme subunit as a tether, and each arm is extended with a single-strand tether for subsequent cross-linking. The strand (**21**) is modified with the green fluorescence label (fluorescein) (10% doping) to follow the dynamic features of the MDs. Subjecting the Y-shaped module **Y5** to the fuel strand (**24**) results in the cross-linking of the toehold domain of the **Y5** arms. The fuel strand sequence is, however, engineered to include a tether composed of the Mg^2+^-ion-dependent DNAzyme subunit that assembles and cooperatively stabilizes the supramolecular DNAzyme-functionalized MDs, **MD5**. The ribonucleobase-functionalized DNA hairpin H_M_, (**25**), is added as a substrate for the DNAzyme catalytic units. The DNAzyme-catalyzed cleavage of the hairpin yields two fragmented metabolite products (H_Ma_) and (H_Mb_). The strand H_Ma_ was, however, pre-engineered to displace the cross-linking fuel-bridging units (**24**). The displaced duplex (**24**)/H_Ma_ separates the MD framework. Thus, the Y-shaped framework **Y5** subjected to the fuel strand (**24**) and the hairpin H_M_ leads to the transient formation of the DNAzyme-modified **MD5** and to their dissipative depletion by the catalytic cleavage of the hairpin H_M_ accompanied by the metabolite-driven separation of the MD frameworks (the gel electrophoretic characterization of the assembly/depletion of the **MD5** framework is displayed in Figure S26). The participation of the DNAzyme-catalyzed cleavage of H_M_ as the key process stimulating the dynamic transient depletion of the MDs was confirmed by substituting the hairpin H_M_, (**25**), with an all-DNA analogue sequence (**26**). Under these conditions of (**26**), the fuel-driven formation of **MD5** was observed, yet their dissipative depletion was blocked. Figure 5B,C depicts the dynamic transient behavior of the MDs by confocal fluorescence microscopy (the fluidic properties of **MD5** are discussed in Figure S27). The MDs dynamically evolve within a time interval of 60 min with sizes of ca. 5 μm, and subsequently, the DNAzyme-driven depletion of the MDs proceeds for a time interval of 180 min (Figure 5C, Panel I). The dynamic formation and depletion of the condensates are controlled by the concentrations of the fuel strand (**24**) and the concentrations of the hairpin (**25**) H_M_ (Figure 5C, Panels II and III). As the concentration of the fuel strand is elevated, the time interval forming the MDs is prolonged, and larger condensates are formed (Panel II). As the concentration of H_M_ increases, the peak sizes of the generated MDs are smaller, and the depletion rates of the condensates are faster (Panel III).

**Figure 6:**
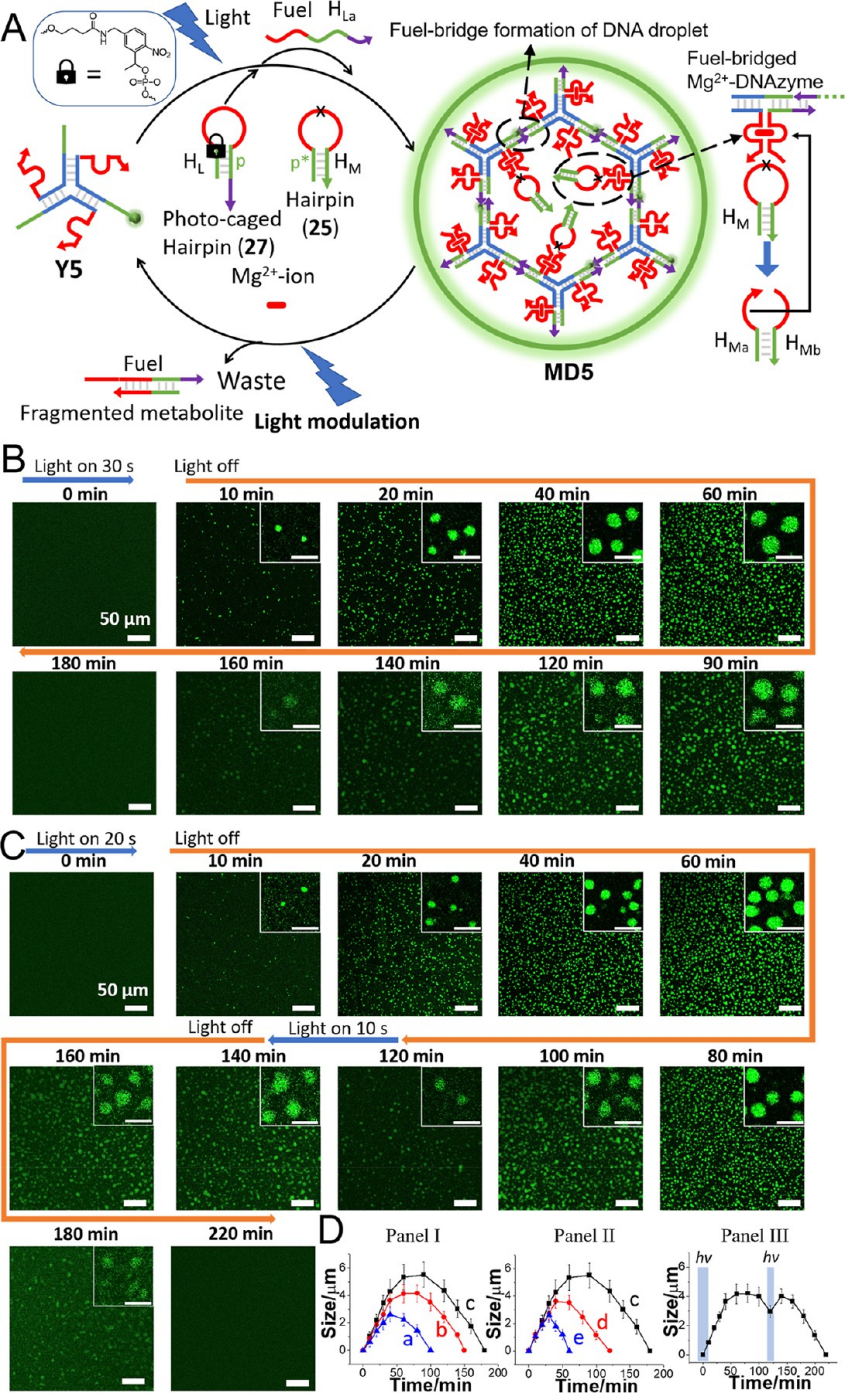
(A) Schematic light-modulated fuel-driven assembly of Mg^2+^-ion-dependent DNAzyme cross-linked MD coacervates **MD5** undergoing, in the presence of a photoresponsive hairpin H_L_ and DNAzyme substrate hairpin H_M_, transient evolution/depletion of the MDs. (B) Confocal fluorescence microscopy images (scale bar = 50 μm, inset scale bar = 10 μm) corresponding to the light-triggered (λ = 365 nm, 100 mW, 30 s) fueled cross-linking of the reaction module **Y5** (8 μM) in the presence of the photoresponsive hairpin H_L_ (24 μM) and H_M_ (18 μM). (C) Confocal fluorescence microscopy images (scale bar = 50 μm, inset scale bar = 10 μm) corresponding to the light-triggered and light-modulated transient evolution/depletion of the MDs. The primary light-triggered activation of the transient formation of the dissipative MDs proceeds by applying a light pulse for 20 s. After a time interval of transient depletion of 120 min, a modulating light pulse of 10 s is applied to refuel the dissipative system. (D) Panel I—Temporal MD sizes following the light-triggered transient evolution depletion of the MDs using different light doses activating the fueled formation of the MDs: (a) 10 s, (b) 20 s, and (c) 30 s. Panel II—Temporal MD sizes corresponding to the light-triggered evolution–depletion of the MDs, illuminated for 30 s, in the presence of variable concentrations of H_M_: (c) 18 μM, (d) 21 μM, and (e) 24 μM. Panel III—Temporal size changes of the MDs upon the light-triggered activation of the MD formation by a light pulse of 20 s, followed by a modulating light pulse of 10 s with the transient depletion of the MDs. (Error bars in all experiments are derived from *N* = 3 experiments, analyzing in each experiment 4 imaged frames.)

The built-in integration of a DNAzyme constituent driving the transient depletion of the MDs suggested, however, that the system could be further developed by introducing an auxiliary trigger to form the dissipative MDs and, particularly, to apply a trigger allowing the temporal modulation of the MD morphologies during their transient depletion. This was accomplished using light as an auxiliary trigger to stimulate the formation of the dissipative coacervate MDs and to modulate their shapes during the dynamic depletion of the MDs, as outlined in Figure 6A. The Y-shaped framework **Y5** that includes in its arms the pre-engineered Mg^2+^-ion-dependent subunit interacts with two hairpin structures H_L_ (**27**) and H_M_ (**25**), resulting in a “mute” inactive reaction circuit. Hairpin H_L_ includes a photoresponsive *o*-nitrobenzyl phosphate ester-caged structure, where the sequence “p” consists of the base sequence allowing the cross-linking of the toehold tethers of the Y-shaped “arms”, extended by the sequence of the second loop area subunit of the Mg^2+^-ion-dependent DNAzyme subunit. The second hairpin H_M_ acts as a DNAzyme substrate and includes a caged sequence “p*” complementary to the DNAzyme subunit “p” of H_L_ and a ribonucleobase-modified loop cleavable by an appropriately engineered Mg^2+^-ion-dependent DNAzyme. The light-triggered and light-modulated formation and depletion of the transient coacervate MDs are displayed in Figure 6A. Light-triggered uncaging of the hairpin H_L_ (λ = 365 nm, *P* = 100 mW) separates the strand H_La_, pre-engineered to act as the fuel strand (**24**) cross-linking the Y-shaped module **Y5**, leading to the phase separation of **MD5**. The time of photochemical uncaging of the hairpin H_L_ controls the content of the fuel strand and consequently the rates and sizes of the resulting evolved MDs. Phase separation of the MDs self-assembles Mg^2+^-ion-dependent DNAzyme subunits anchored to the cross-linked units, thus resulting in structurally integrated DNAzyme units in the MD frameworks. The self-assembled DNAzyme units cleave, however, the substrate hairpin H_M_, leading to the generation of the metabolite fragment strand H_Ma_, displacing the cross-linking units, and to the depletion of the catalytically active MDs. That is, the short light-triggered uncaging of H_L_ yields the fuel strand driving the dynamic evolution of the DNAzyme-modified coacervate **MD5**, and the assembly of catalytic Mg^2+^-ion-dependent DNAzyme subunits stimulates the concomitant catalytic cleavage of the hairpin, H_M_, leading to the dynamic transient formation and metabolite-driven depletion of the MDs. Nevertheless, as the photoresponsive hairpin H_L_ is present in excess as a dormant constituent within the dynamic depletion of the MDs, pulsed irradiation of the system allows the light-triggered supply of the fuel strand, H_La_, reactivating the temporal reassembly of the catalytic MDs, thereby leading to the light-modulated temporal size changes of the coacervate MDs. Figure 6B depicts the light-triggered assembly of the DNAzyme-modified **MD5** and their transient depletion through the “dark” catalyzed cleavage of H_M_. At time *t* = 0, the system exists in a mute inactive state. Illumination of the system for 30 s activates the evolution and temporal growth of the MDs that reach an average size of about 5.3 μm at *t* = 60 min. After this time interval, the MDs are temporally depleted through the competitive DNAzyme-catalyzed cleavage of the hairpin H_M_, leading to the separation of the cross-linked coacervates. This is reflected by a temporal decrease in the MD sizes and a lower content of condensates. After a time interval of 180 min, the MDs are fully depleted. The kinetics associated with the phototriggered evolution of the droplets is controlled by the time interval employed to phototrigger the unlocking of H_L_, dictating the dose of fuel, and the concentration of the hairpin substrate H_M_. As the phototriggered unlocking of H_L_ is prolonged, the evolution of the MDs is enhanced, and as the concentration of H_M_ increases, the depletion rate is faster (Figure 6D, Panels I and II). Figure 6C depicts the light-triggered evolution and light-modulated dynamic transient formation and depletion of the MDs. In this experiment, the reaction module is activated with an illumination pulse (λ = 365 nm) for 20 s. The evolution of the DNAzyme-functionalized droplets and their size enlargement proceed for 60 min, reaching the size of ca. 4.1 μm, and afterward, the DNAzyme-mediated depletion is observed, yielding after 120 min the MDs revealing an average size of ca. 2.9 μm. At this time interval, the MDs are subjected to a light pulse (λ = 365 nm) of 10 s duration, resulting in the cleavage of H_L_. Fueling the transient depletion assembly results in the temporal evolution of the coacervate MDs, reflected by their enlargement after 140 min to MDs revealing an average size of 4 μm that afterward decay to smaller MDs that are fully depleted after ca. 220 min. Figure 6D, Panel III depicts the dynamic light-triggered and light-modulated temporal size changes of the MDs, upon the transient formation and dissipative depletion of the MDs. Thus, the catalytic coacervate MDs demonstrate, in the presence of the hairpin substrate H_M_, light-modulated morphological size changes along with their dynamic evolution and dissipative depletion. In fact, these light-modulated dynamic transitions of the coacervate DNAzyme MD protocells emulate dynamic temporal shape transitions of native cells driven across metabolic cycles. (For the concomitant light-triggered modulated evolution and subsequent depletion of the MDs, see Figure S28 and accompanying discussion.)

## Conclusions

The study introduced functional DNA frameworks evolving, in the presence of appropriately engineered DNA fuel strands or duplexes and auxiliary enzymes, phase-separated coacervate MDs undergoing transient, dissipative depletion. Endonucleases or nickases were employed as auxiliary catalysts to transiently deplete the MDs. The MDs may act as protocell containments for the encapsulation of loads (e.g., drugs). The biocatalytic temporal depletion of the MDs may, then, be envisaged as a versatile means for the temporal release of the loads. By using mixtures of Y-shaped frameworks cross-linked by different enzyme-responsive bridging units, gated dissipative formation/depletion of MDs occurs. These results provide versatile means to control the temporal stabilities and dissipative depletion of mixtures of coacervates by means of auxiliary biocatalysts, thereby providing programmable control over concentration, sizes, and load release capacities from synthetic organelles. For example, by tailoring a mixture of three Y-shaped framework/fuel strands responsive to three different endonucleases, the relative temporal sizes, depletion efficacies, and load release of the evolved MDs can be tuned by the relative fuel/biocatalyst concentrations. Furthermore, subjecting the mixture of reaction modules to appropriate inhibitors allows the targeted control over the sizes and load release capacities by the temporal behavior of the coacervates. Moreover, a major advance in the fabrication of transient coacervate is demonstrated by the integration of DNAzyme units in the phase-separated frameworks, generating functional MDs exhibiting self-programmable shape morphologies. This approach might be extended to engineer other integrated functional stimuli-responsive MDs, such as aptamer/ligand- or pH- or G-quadruplex-responsive MDs. These MDs could be engineered to operate temporal transformations of enhanced complexities through the cleavage of appropriately designed hairpin substrates, where their cleaved metabolite products act as information transfer strands providing a means to evolve branched coacervate MDs revealing tailored functionalities. For example, the cleaved hairpin products could act as fuel strands, generating cascaded or branched DNAzyme-triggered transient MD coacervates. Such a system could add to the “parent” MD protocell the element of dynamic proliferation capacities. Moreover, we introduce the use of photoresponsive hairpin structures as functional constituents for the light-triggered evolution of functional MDs and for light-modulated control over the temporal shape morphologies. The time-dependent stimuli-triggered, metabolite-controlled dynamic shape changes of the phase-separated catalytic coacervates emulate dynamic cellular shape changes during metabolic cycles, thus providing synthetic protocell frameworks.

## Supporting Information Available

Experimental section; gel electrophoretic experiment of **MD1**; transient assembly/depletion of **MD2**; specificity of **MD1** and **MD2** to the endonuclease; sequence parameters affecting the dynamics of endonuclease-responsive MD evolution; tunable transient operation of nongated and inhibitor-gated **MD1** and **MD2** coacervate mixtures; orthogonal assembly of **MD1** and **MD2**; assembly/depletion of **MD3**; transient assembly/depletion of **MD4**; specificity of **MD3** and **MD4** to nickase; orthogonal, selective transient assembly/depletion of **MD3** and **MD4**; evaluation of energy input/output values associated with the different dissipative MD systems; catalytic rate estimation of the endonucleases/nickases in the different MDs; assembly/depletion of Mg^2+^-ion DNAzyme-modified **MD5**; light-modulated transient assembly/depletion of **MD5** (PDF)
