# Supplementary material for: Enzyme- and DNAzyme-Driven Transient Assembly of DNA-Based Phase-Separated Coacervate Microdroplets
Source: J Am Chem Soc. 2025 Apr 30;147(19):16141–53. doi: 10.1021/jacs.5c00637 (PMC12082633; doi:10.1021/jacs.5c00637)
Supplement: Supplementary file 1 — ja5c00637_si_001.pdf [file ja5c00637_si_001.pdf]

Supporting information for

Enzyme- and DNAzyme-Driven Transient Assembly of DNA-Based Phase-Separated Coacervate Microdroplets

Yunlong Qin,[a] *Yang Sung Sohn,*[b] *Rachel Nechushtai,*[b] *Fan Xia,*[c] *Fujian Huang,*[c]* *and Itamar Willner* [a]*

[a]The Institute of Chemistry, The Hebrew University of Jerusalem, Jerusalem 91904, Israel. Email: itamar.willner@mail.huji.ac.il.

[b]The Institute of Life Science, The Hebrew University of Jerusalem, Jerusalem 91904, Israel.

[c]State Key Laboratory of Biogeology and Environmental Geology, Faculty of Materials Science and Chemistry, China University of Geosciences, Wuhan 430074, China. Email: huangfj@cug.edu.cn.

Contents

Experimental section ... 3
Materials ... 3
Characterization ... 4
Endonuclease-controlled transient formation/depletion of MD1 or MD2 ... 5
Endonuclease-dictated Orthogonal, Gated, or Selective transient assembly/depletion of green fluorescent MD1 and red fluorescent MD2 ... 5
Nickase-controlled transient formation/depletion of MD3 or MD4 ... 6
Nickase-dictated Orthogonal or Selective transient assembly/depletion of green fluorescent MD3 and red fluorescent MD4 ... 7
Transient assembly/depletion of Mg^{2+}-ion-dependent DNAzyme-functionalized phase-separated MD5 ... 7
Light-triggered and Light-modulated transient assembly/depletion of Mg^{2+}-ion-dependent DNAzyme-functionalized phase-separated MD5 ... 8
Fluidic nature of phase-separated microdroplets MD1 ... 9
Assembly/depletion of endonuclease (EcoRI)-responsive microdroplets MD1: gel electrophoresis experiments ... 10
Transient formation/depletion of endonuclease (HindIII)-responsive microdroplets MD2 ... 11
Parameters affecting the dynamics of endonuclease-responsive microdroplets evolution ... 17
Tunable transient operation of the Non-Gated or inhibitor-Gated MD1/MD2 coacervates mixtures ... 20
Specificity of MD1 and MD2 in the presence of a foreign endonuclease ... 24
Orthogonal formation of phase-separated MD1 and MD2 ... 26
Assembly/depletion of nickase (Nt.BbvCI)-responsive phase-separated microdroplets MD3 ... 28
Transient assembly/depletion of nickase (Nb.BtsI)-responsive phase-separated microdroplets MD4 ... 31
Specificity of MD3 and MD4 in the presence of a foreign nickase ... 36
Orthogonal, and selective transient formation/depletion of phase-separated MD3 and MD4 ... 38
Evaluation of the energy input/output values associated with the different enzyme-driven dissipative microdroplet system ... 43
Estimation of the catalytic rates of the endonucleases/nickases in the different MDs ... 49
Assembly/depletion of Mg^{2+}-ion-dependent DNAzyme-modified phase-separated microdroplets MD5 ... 50
Light-modulated transient formation/depletion of Mg^{2+}-ion-dependent DNAzyme-modified MD5 ... 53

Experimental section

Materials

Trizma base (Tris), , hydrochloride (HCl, 37%), magnesium acetate ($Mg(OAc)_2$), potassium acetate (KOAc) were purchased from Sigma-Aldrich. Endonuclease (EcoRI and HindIII), Nickase (Nt.BbvCI and Nb.BtSI), rCutsmart buffer (10x) were obtained from New England Biolabs. GelRed (10000x) was obtained from Biotium. Ultrapure water was obtained by a NANOpure Diamond apparatus. The DNA strands were ordered from Integrated DNA Technologies and listed below.

(**1**): 5’-CATGTCACCACGCTGTCCTAACTTCATGACCGTCGAAG-3’

(**1-FAM**): 5’-FAM-CATGTCACCACGCTGTCCTAACTTCATGACCGTCGAAG-3’

(**2**): 5’-CATGTCACCTTCGACGGTCATGTTTACTAGATCAGAGG-3’

(**3**): 5’-CATGTCACCCTCTGATCTAGTATTGTTAGGACAGCGTG-3’

(**4**): 5’-TGACATGCATGAGAATTCCATTC -3’

(**5**): 5’-TGACATGGAATGGAATTCTCATG-3’

(**6**): 5’-TGAGGTTTCACGCTGTCCTAACTTCATGACCGTCGAAG-3’

(**6-Cy5**): 5’-Cy5-TGAGGTTTCACGCTGTCCTAACTTCATGACCGTCGAAG-3’

(**7**): 5’-TGAGGTTTCTTCGACGGTCATGTTTACTAGATCAGAGG-3’

(**8**): 5’-TGAGGTTTCCTCTGATCTAGTATTGTTAGGACAGCGTG-3’

(**9**): 5’-AACCTCACATGAAAGCTTCATTC-3’

(**10**): 5’-AACCTCAGAATGAAGCTTTCATG-3’

(**11**): 5’-AAACCTCA-3’

(**12**): 5’-GTGACATG-3’

(**13**): 5’-TTGCTGAGGTTTCTTCCTGAACGTGTTCTTCCTAATGATGCTCGTG-3’

(**13-FAM**): 5’-FAM-TTGCTGAGGTTTCTTCCTGAACGTGTTCTTCCTAATGAT-GCTCGTG-3’

(**14**): 5’-TTGCTGAGGTTTCACGAGCATCATTAGGTTGTTGACGACATCCATG-3’

(**15**): 5’-TTGCTGAGGTTTCATGGATGTCGTCAACTTGAACACGTTCAGGAA-G-3’

(**16**): 5’-AAACCTCAGCAATTGGCCA-3’

(**17**): 5’-GCAGTGAGCTTTCTTCCTGAACGTGTTCTTCCTAATGATGCTCGTG-3’

(**17-Cy5**): 5’-Cy5-GCAGTGAGCTTTCTTCCTGAACGTGTTCTTCCTAATGATG-GTG-3’

(**18**): 5’-GCAGTGAGCTTTCACGAGCATCATTAGGTTGTTGACGACATCCATG-3’

(**19**): 5’-GCAGTGAGCTTTCATGGATGTCGTCAACTTGAACACGTTCAGGAA-G-3’

(**20**): 5’-AAAGCTCACTGCAACCGGT-3’

(**21**): 5’-TTCTAGGGTTAGCTTCCTGAACGTGTTCTTCCTAATGATGCTCGTGA-CAACGAATACTA-3’

(**21-FAM**): 5’-FAM-TTCTAGGGTTAGCTTCCTGAACGTGTTCTTCCTAATGAT-GCTCGTGACAACGAATACTA-3’

(**22**): 5’-TTCTAGGGTTAGCACGAGCATCATTAGGTTGTTGACGACATCCATG-ACAACGAATACTA-3’

(**23**): 5’-TTCTAGGGTTAGCATGGATGTCGTCAACTTGAACACGTTCAGGAAG-ACAACGAATACTA-3’

(**24**): 5’-GACATGAGGCTAGCTCTAACCCTAGAATTGGCCA-3’

(**25**): 5’-GGGCCAATTCTAGGGTTAGTTAGCTAGTAT**rGrU**CATGTCCTTCTAA-CCCTAGAATTGGCCC-3’

(**26**): 5’-GGGCCAATTCTAGGGTTAGTTAGCTAGTATGTCATGTCCTTCTAACC-CTAGAATTGGCCC-3’

(**27**): 5’-TTCTAGG/PC/GTTAGAG/PC/GACATGAGGCTAGCTCTAACCCTAGA-ATTGGCCA-3’

Characterization

Ultraviolet-visible (UV-vis) spectra were recorded with a UV-2450 spectrophotometer (Shimadzu) to calibrate the concentrations of DNA strands. Gel electrophoresis were operated using a vertical electrophoresis system (Cleaver Scientific, omniPAGE Mini). Confocal fluorescence microscopy images were recorded

using an Olympus FV3000 confocal laser-scanning microscope.

Endonuclease-controlled transient formation/depletion of MD1 or MD2

For the preparation of Y-shaped DNA module **Y1** and fuel duplex (**4**)/(**5**) equimolar concentration (20 μM) of constitutional strand (**1**), (**2**), (**3**) and equimolar concentration (30 μM) of (**4**), (**5**) were separately mixed in two PCR tubes, to which 10 mM of Tris-HCl buffer (pH = 8) and 20 mM KOAc were added. It should be noted that, component (**1**) was mixed with 10% of FAM-labeled strand (**1-FAM**). Annealing of the mixture at 95 ℃ for 5 min followed by its cooling to 25 ℃ over 2 h generated the Y-shaped DNA module **Y1** and fuel duplex (**4**)/(**5**). Immediately after the cooling of the Y-shaped DNA module **Y1** and fuel duplex (**4**)/(**5**), the DNA modules was further treated with rCutsmart buffer (1x, containing 50 mM KOAc, 20 mM Tris-acetate, 10 mM $Mg(OAc)_2$, 100 μg/ml Recombinant Albumin, pH 7.9@25°C), which is necessary for the endonuclease or nickase activity. (Note that the rCutsmart buffer was added after the annealing to avoid possible inactivation of the buffer solution by annealing process.)

To assemble the phase-separated **MD1**, 10 μM of Y-shaped DNA module **Y1** and 15 μM of fuel (**4**)/(**5**) were mixed in a sealed glass chamber (10 μL totally), and stay static at 25 ℃ to allow the MDs to grow. The sample was imaged by confocal fluorescence microscopy at different time intervals. The transient dissipative evolution/depletion of **MD1** was conducted in a similarly way in the presence of endonuclease EcoRI.

The preparation of Y-shaped module **Y2** and fuel duplex (**9**)/(**10**), the assembly of phase-separated **MD2**, and the transient evolution/depletion of **MD2** was conducted in a similar way as stated above regarding **MD1**, except the concentrations of DNA modules and endonuclease HindIII.

All experiments related with endonuclease-responsive MDs **MD1** or **MD2** were conducted at 25 ℃.

Endonuclease-dictated Orthogonal, Gated, or Selective transient assembly/depletion of green fluorescent MD1 and red fluorescent MD2

The Y-shaped module **Y1** and **Y2** were annealed separately using a similar method

as above mentioned, but using the constituents at a concentration of 40 μM, and the fuel (**4**)/(**5**) and (**9**)/(**10**) were annealed separately using the components at a concentration of 60 μM.

For the orthogonal assembly of green fluorescent **MD1** and red fluorescent **MD2**, 15 μM of (**4**)/(**5**) and 9 μM of (**9**)/(**10**) were subjected to a mixture of 10 μM of **Y1** module and 6 μM of **Y2** module, followed by the static incubation at 25 °C allowing the orthogonal growth of the **MD1** and **MD2**.

To perform the transient (gated) orthogonal assembly/depletion of **MD1** and **MD2**, the mixture of fuel (**4**)/(**5**) and (**9**)/(**10**) were subjected to the mixture of **Y1** module, **Y2** module, and two endonucleases, EcoRI and HindIII, without inhibitors (**Non-Gated**), in the presence of inhibitor (**11)** (18 μM) (**Gated-1**) or in the presence of inhibitor (**12**) (30 μM) (**Gated-2**). The concentration of endonucleases in the experiments are adjusted and specified accordingly.

The selectively transient assembly/depletion of **MD1** and **MD2** was performed by subjecting the mixture of fuel (**4**)/(**5**) and (**9**)/(**10**), to a mixture **Y1** module and **Y2** module, in the presence of a single endonuclease, EcoRI or HindIII.

All experiments above were conducted at 25 °C.

Nickase-controlled transient formation/depletion of MD3 or MD4

The Y-shaped module **Y3** was prepared by annealing the mixture of components (**13**), (**14**), and (**15**) with equal molar concentrations (10 μM), in the presence of 10 mM of 10 mM of Tris-HCl buffer (pH = 8) and 20 mM KOAc, at 95 °C for 5 min followed by its cooling to 25 °C over 2 h. Then, rCutsmart buffer (1x) was added to the mixture for further experiments. (Note that strand (**13**) was doped with 10% of fluorophore-labeled strand (**13-FAM**).)

To assemble the phase-separated microdroplets **MD3**, 30 μM of fuel strand (**16**) with palindromic tethers was subjected to the Y-shaped module **Y3**, followed by the static incubation at 23 °C to allow the growth of the MDs. The samples were imaged by confocal fluorescence microscopy at different time intervals. The transient assembly/depletion of the **MD3** were conducted in the presence of nickase Nt.BbvCI.

The preparation of Y-shaped module **Y4**, the assembly of phase-separated **MD4**, and the transient evolution/depletion of **MD4** was conducted in a similar way as **MD3**, except using the nickase Nb.BtsI.

All experiments were conducted at 23 °C.

Nickase-dictated Orthogonal or Selective transient assembly/depletion of green fluorescent MD3 and red fluorescent MD4

The Y-shaped module **Y3** and **Y4** were prepared at a concentration of 20 μM, followed by the addition of rCutsmart buffer (1x) and extra 10 mM of $Mg(OAc)_2$. For the orthogonal formation of **MD3** and **MD4**, a mixture of fuel (**16**) and fuel (**20**) (each 30 μM) was added to the mixture of Y-shaped module **Y3** and **Y4** (each 10 μM). For the orthogonal or selective transient formation/depletion of **MD3** and **MD4**, the mixture of fuel (**16**) and (**20**) was subjected to the mixture of **Y3** and **Y4** in the presence of two nickases (Nt.BbvCI and Nb.BtsI), one nickase (Nt.BbvCI), or alternatively, another nickase (Nb.BtsI).

All experiments were conducted at 23 °C.

Transient assembly/depletion of Mg^{2+}-ion-dependent DNAzyme-functionalized phase-separated MD5

The Y-shaped module **Y5** was prepared by annealing of the components (**21**), (**22**), and (**23**) with a concentration of 10 μM each, in the presence of 10 mM Tris-HCl buffer (pH = 8), 50 mM KOAc, and 30 mM $Mg(OAc)_2$, at 95 °C for 5 min followed by its cooling to 25 °C over 2 h. Note that strand (**21**) was doped with 10% of fluorophore-labeled strand (**21-FAM**).

To prepare metabolite H_M, 100 nM of strand (**25**) was dispersed in 50 mL of 10 mM Tris-HCl buffer (pH = 8), 50 mM KOAc, and 30 mM $Mg(OAc)_2$. The mixture was separated into aliquots in 1.5 mL Eppendorf tube, followed by the annealing in a heating block at 95 °C for 15 min. Immediately, the samples were subjected to ice cooling for 10 min, and afterwards incubated at room temperature for 1 h to from the well-caged hairpin structure, H_M. The 100 nM of H_M was then concentrated to 50 μM ~100 μM using Amicon Ultra Centrifugal Filters (3000 Da, 15 mL and 3000 Da, 0.4 mL,

separately). The concentration of hairpin H_M was evaluated by the extinction coefficient at 260 nm of the strand.

For the typical transient assembly/depletion of the Mg^{2+}-ion-dependent DNAzyme-modified MDs **MD5**, the fuel strand (**24**) (24 μM) was subjected to the mixture of Y-shaped module **Y5** (8 μM) and metabolite H_M (24 μM, 21 μM, or 18 μM). The sample was incubated statically at 27 °C, and imaged by confocal fluorescence microscopy at different time intervals.

Light-triggered and Light-modulated transient assembly/depletion of Mg^{2+}-ion-dependent DNAzyme-functionalized phase-separated MD5

The Y-shaped DNA module **Y5** and metabolite H_M was prepared similarly as above mentioned. The photocaged hairpin H_L was prepared by annealing of 96 μM strand (**27**) at 95 °C for 5 min, followed by cooling to 4 °C for 4 min, and finally kept at 25 °C for 1 h.

For typical light-triggered transient formation/depletion of Mg^{2+}-ion-dependent DNAzyme-functionalized phase-separated **MD5**, the Y-shaped module **Y5** (24 μM), and metabolite H_M (18 μM) was mixed with the photocaged hairpin H_L (24 μM), which generated the fragmented H_{La} acting as fuel (**24**). The mixture was then subjected to light illumination (365 nm, 100 mW) for 10 s, 20 s, or 30 s, to trigger the transient formation/depletion of **MD5**.

For light-modulated transient formation /depletion of Mg^{2+}-ion-dependent DNAzyme-functionalized phase-separated **MD5**, the above mixture of samples was subjected to light illumination for short pulses separately as mentioned in the main text.

All experiments were conducted at 27 °C.

Fluidic nature of phase-separated microdroplets MD1

The phase-separated MDs **MD1** exhibits a fluidic, liquid-like, dynamically-exchangeable constituents property, which was reflected by the fluorescence recovery after photobleaching (FRAP) experiment, Figure 1(C). As shown in Figure 1(C), Panel I, the upper half domain of a single **MD1**, as the bleaching region of FRAP, was exposed to an intensified confined laser for 2 s at t = 10 s, resulting in a totally FAM-bleached **Y1** constituents in the upper domain. Afterwards, the single MD was imaged at time intervals to observe the fluorescence recovery process. At t = 120 s, the fluorescence upper bleached domain is almost fully recovered, and the recovered fluorescence originated from the non-bleached **Y2** module from the lower half domain of the MD, demonstrating the fluid, dynamically exchangeable properties of the DNA modules in **MD2**. Figure 1(C), Panel II showed the temporal normalized fluorescence intensities in the upper bleached domain of the single MD, the short recovery time of ca. 120 s indicated the fluid-like properties in the **MD2** containment, instead of gel-like properties.

Assembly/depletion of endonuclease (EcoRI)-responsive microdroplets MD1: gel electrophoresis experiments

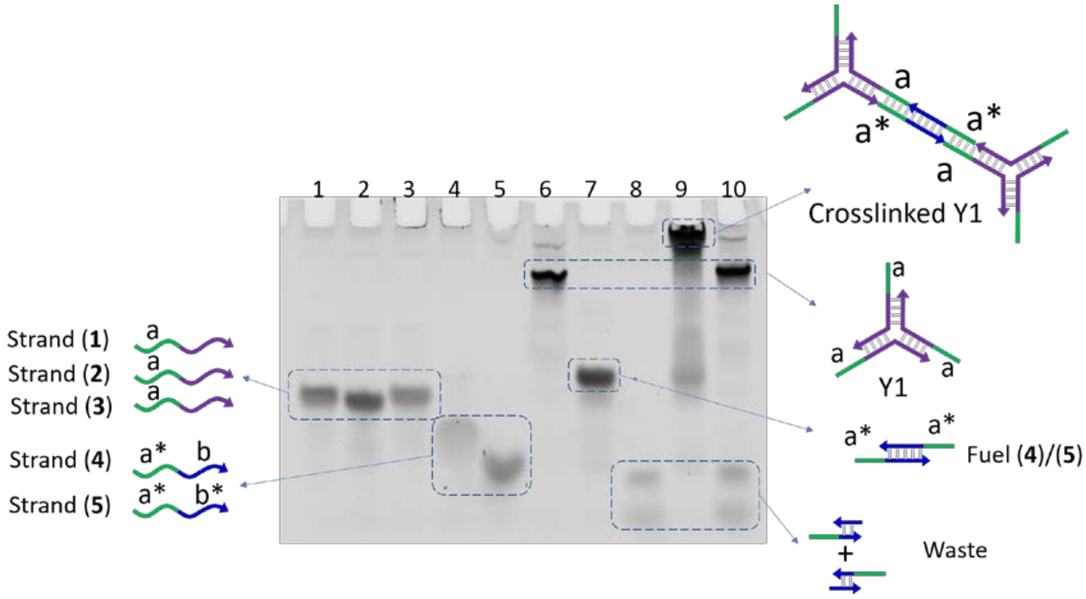

Figure S1. Gel electrophoresis analysis of the assembly of endonuclease EcoRI-responsive **MD1**. Lane 1: strand (**1**); lane 2: strand (**2**); lane 3: strand (**3**); lane 4: strand (**4**); lane 5: strand (**5**); lane 6: **Y1** = (**1**)/(**2**)/(**3**); lane 7: Fuel (**4**)/(**5**); lane 8: Fuel (**4**)/(**5**) + EcoRI; lane 9: **Y1** + Fuel (**4**)/(**5**); lane 10: **Y1** + Fuel (**4**)/(**5**) + EcoRI.

The assembly and separation of EcoRI-dictated microdroplets **MD1** was also supported by gel electrophoretic experiment, Figure S1. The assembly of Y-shaped module **Y1** and fuel (**4**)/(**5**) are displayed in the band of lane 6 and lane 7, respectively, as compared to the band of constitutional strands in lane 1 ~ lane 5. Subjecting the EcoRI-cleavable fuel duplex (**4**)/(**5**) to the EcoRI endonuclease led to the band of fragmented wastes in lane 8. The incubation of the Y-shaped module **Y1** with the fuel duplex (**4**)/(**5**) led to the crosslinked framework **MD1** corresponding to lane 9. Treatment of the Y-shaped module **Y1**, the duplex (**4**)/(**5**), and EcoRI led to the bands displayed in lane 10, where only traces of **MD1** are observed, and the bands of released **Y1** and waste products are visible, consistent with the transient depletion of **MD1**.

Transient formation/depletion of endonuclease (HindIII)-responsive microdroplets MD2

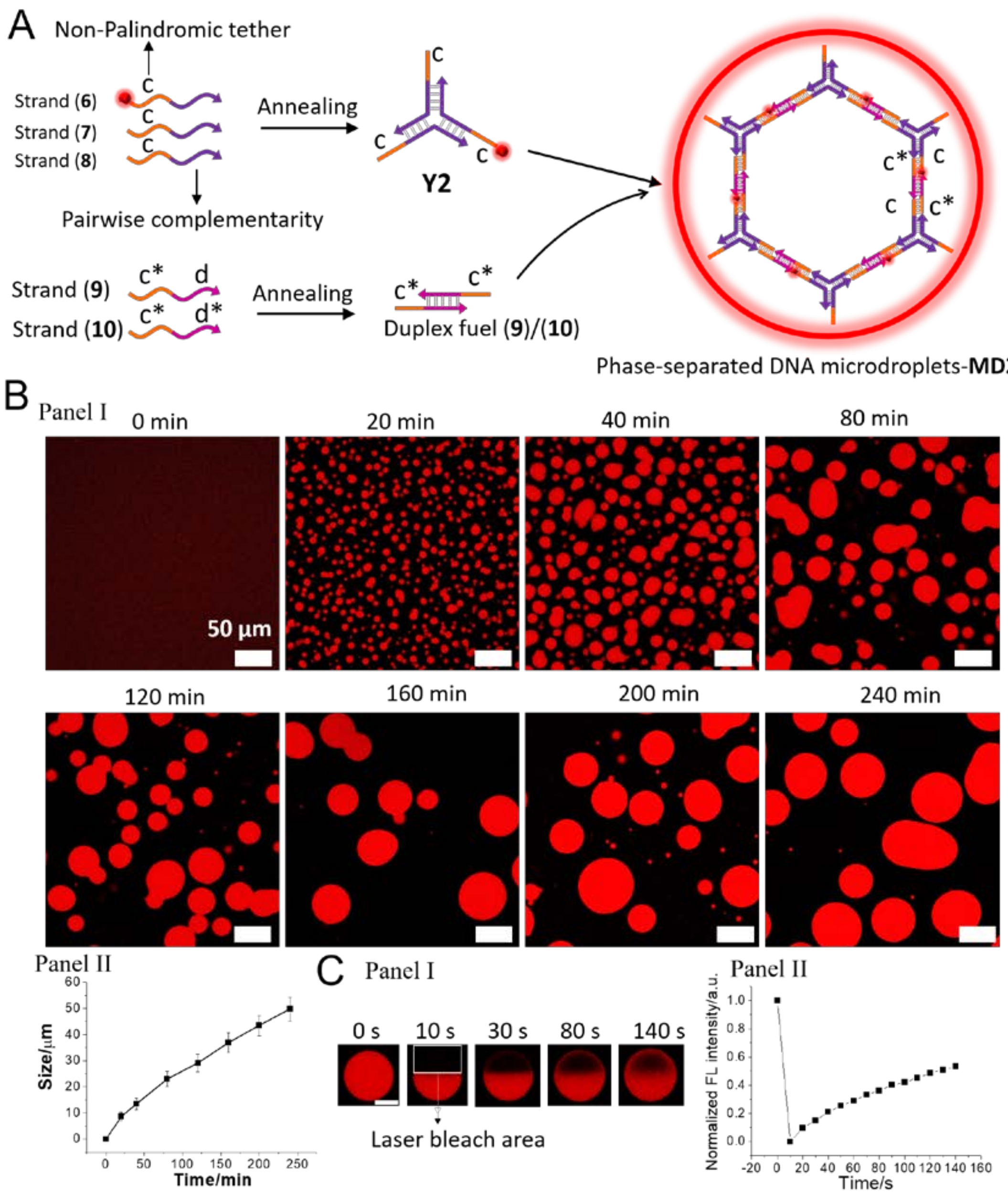

Figure S2. (A) Schematic assembly of a second endonuclease HindIII-responsive **MD2** by the crosslinking of Cy5-labeled Y-shaped module **Y2** with HindIII-cleavable fuel duplex (**9**)/(**10**). (B) Panel I-Temporal confocal fluorescence microscopy images (scale bar = 50 μm) of phase-separated microdroplets **MD2**. Panel II-Average size changes corresponding to the temporal growth of **MD2**. **Y2** = 6 μM, and (**9**)/(**10**) = 9μM. (C) Panel I-Temporal confocal fluorescence microscopy images (scale bar = 5 μm) of a single **MD2** corresponding to the fluorescence recovery after photobleaching (FRAP) in the upper domain of the microdroplet. Panel II-Temporal normalized fluorescence intensity changes in the FRAP domain of the single microdroplet.

The endonuclease-dictated cleavage of specific base sequence in DNA duplex was, then applied to design a second type of HindIII-driven transient assembly/depletion of Cy5-labeled MDs **MD2**, Figures S2 ~ S4. The assembly of the MDs **MD2** was depicted in Figure S2(A). The annealing of a mixture of components (**6**)/(**7**)/(**8**) and (**9**)/(**10**) separately generated the Y-shaped module **Y2** with arm-extended free toehold tethers c, and fuel duplex (**9**)/(**10**) with extended free tethers c*, where c* was pre-engineered to be hybridized with free c toehold tethers. (Note that 10% of strand (**6**) was labeled with a red-fluorescent Cy5 fluorophore.) Accordingly, subjecting appropriate amount fuel duplex (**9**)/(**10**) to Y-shaped module **Y2** generated c/c* crosslinked hexagonal framework, yielding the red-fluorescent phase-separated DNA microdroplets **MD2**. Figure S2(B), Panel I displayed the temporal confocal fluorescence microscopy images of phase-separated MDs **MD2**, revealing dynamic growth features. The statistical average sizes of **MD2** from confocal fluorescence microscopy images was depicted in Figure S2(B), Panel II. The phase-separated MDs **MD2** revealed a similar size distribution of ca. 30 μm within a time interval of 160 min. The MDs exhibits a fluidic, liquid-like, dynamically-exchangeable constituents property in the containment, which was demonstrated by the fluorescence recovery after photobleaching (FRAP) experiment, Figure S2(C). As shown in Figure S2(C), Panel I, the upper domain of a single MD was chosen as the bleach region of FRAP, where an intensified confined laser was applied for 2 s at t = 10 s, resulting in a totally Cy5-bleached DNA constituents in the upper domain. Afterwards, the single MD was imaged at time intervals to observe the fluorescence recovery process. At t = 140 s, the upper bleached domain is partly

recovered, where the recovered fluorescence originated from the non-bleached DNA Y-shaped module **Y2** from the lower domain of the MD, demonstrating the fluid, dynamically exchangeable properties of the DNA Y-shaped module in **MD2**. Figure S2(C), Panel II showed the temporal normalized fluorescence intensities in the upper bleached domain of the single MD, the short recovery time of ca. 140 s indicated the fluid-like properties in the **MD2** containment, instead of gel-like properties.

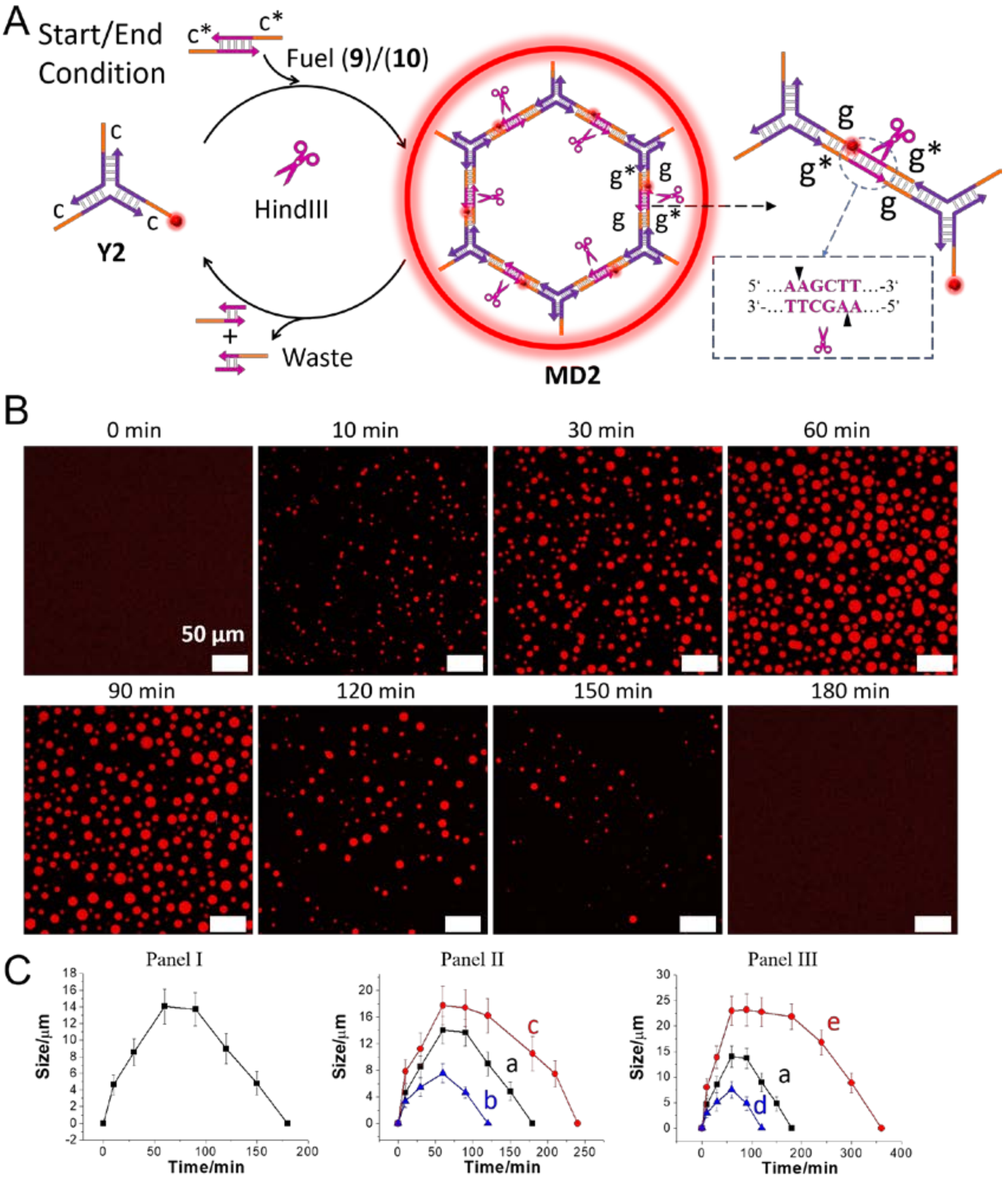

Figure S3. (A) Schematic transient formation/depletion of a second endonuclease HindIII-controlled microdroplets **MD2** by the fueled crosslinking of Cy5-labeled **Y2** with HindIII-cleavable fuel duplex (**9**)/(**10**) in the presence of HindIII. (B) Temporal confocal fluorescence microscopy images (scale bar = 50 μm) of transient dissipative formation/depletion of phase-separated microdroplets **MD2**. (C) Panel I-Temporal average size changes of phase-separated **MD2**, in the presence of **Y2**, 6 μM, fuel (**9**)/(**10**), 9 μM, and HindIII, 8 U/μL. Panel II-Temporal average size changes of phase-separated **MD2**, in the presence of **Y2**, 6 μM, and HindIII, 8 U/μL, and variable concentrations of fuel (**9**)/(**10**): (a) 9 μM, (b) 7.5 μM, (c) 10.5 μM. Panel III-Temporal average size changes of phase-separated **MD2**, in the presence of **Y2**, 6 μM, and fuel (**9**)/(**10**), 9 μM., and variable concentrations of HindIII: (a) 8 U/μL, (d) 12 U/μL, (e) 4 U/μL.

The transient, dissipative formation and depletion of phase-separated MDs **MD2** was schematically displayed in Figure S3(A). Subjecting the fuel duplexes (**9**)/(**10**) to a mixture of Y-shaped module **Y2** and HindIII endonuclease, led to the crosslinking of c/c* tethers associated with **Y2** and fuel (**9**)/(**10**) respectively, resulting in the formation of phase-separated **MD2**. The fuel (**9**)/(**10**) was, however, pre-engineered to be specifically recognized and cleaved by HindIII endonuclease, leading to the concomitant digestion of the fuel (**9**)/(**10**) and crosslinked framework. The concomitant cleavage of crosslinking unit (**9**)/(**10**) limited the growth of phase-separated **MD2**, and ultimately depleted all the MDs, regenerating the solution-dispersed Y-shaped module **Y2** with the generation of cleaved waste products. The transient time-dependent growth and depletion of the **MD2** was probed by the confocal fluorescence microscopy images, Figure S3(B). Within ca. 60 min, the growth of the **MD2** proceeded, reaching a maximum average size of 12 μm. The **MD2** started to be degraded after 90 min, reflected by the less content of MDs and decreased average sizes, and was fully depleted after 180 min. The temporal size changes of the **MD2** was depicted in Figure S3(C) Panel I, and the sizes and transient depletion time of **MD2** are controlled by the concentration of fuel (**9**)/(**10**) and the concentration of HindIII endonuclease, Figure S3(C) Panel II, and Panel III, respectively. As the concentration of fuel (**9**)/(**10**) increases, the maximum growth size of **MD2** is larger and dissipative depletion time is longer. As the concentration of HindIII increases, the peak growth size of **MD2** is smaller with a shorter transient depletion time.

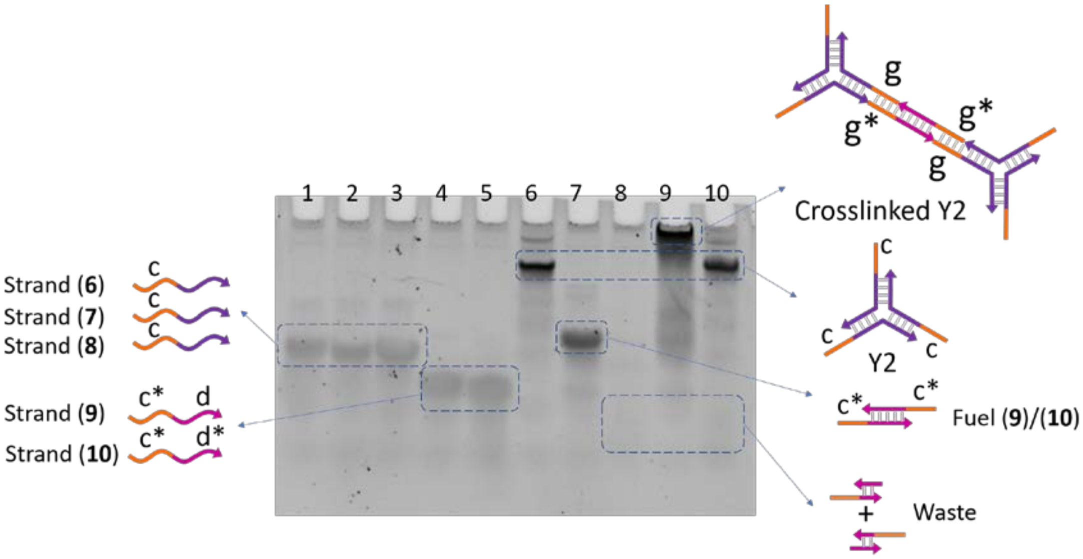

Figure S4. Gel electrophoresis analysis of the assembly of endonuclease HindIII-responsive **MD2**. Lane 1: strand (**6**); lane 2: strand (**7**); lane 3: strand (**8**); lane 4: strand (**9**); lane 5: strand (**10**); lane 6: **Y2** = (**6**)/(**7**)/(**8**); lane 7: Fuel (**9**)/(**10**); lane 8: Fuel (**9**)/(**10**) + HindIII; lane 9: **Y2** + Fuel (**9**)/(**10**); lane 10: **Y2** + Fuel (**9**)/(**10**) + HindIII.

The assembly/depletion of endonuclease HindIII-controlled microdroplets **MD2** was also supported by gel electrophoresis experiment, Figure S4. The assembly of Y-shaped module **Y2** and fuel (**9**)/(**10**) was confirmed in the band of lane 6 and lane 7, respectively, as compared to the band of constitutional strands in lane 1 ~ lane 5. Digestion of the endonuclease HindIII-cleavable fuel duplex (**9**)/(**10**) by HindIII resulting in the band of fragmented wastes in lane 8. The incubation of the Y-shaped module **Y2** with the fuel duplex (**9**)/(**10**) led to the crosslinked framework of **MD2** in lane 9. Treatment of the HindIII-responsive **MD2** framework with HindIII, led to the bands in lane 10, recovering the band the Y-shaped module **Y2** and generating the band of waste.

Parameters affecting the dynamics of endonuclease-responsive microdroplets evolution

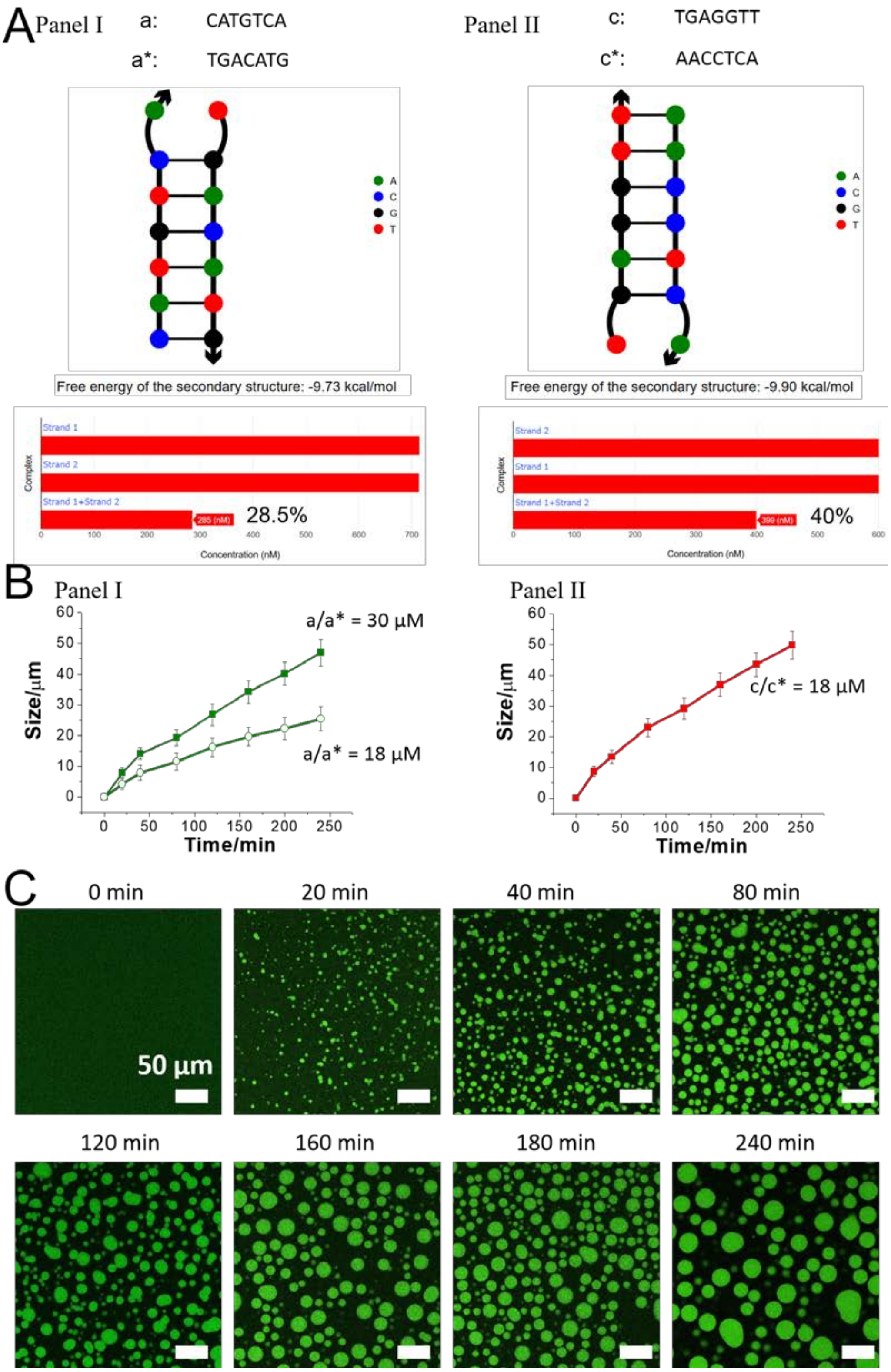

Figure S5. (A) The computational thermodynamic evaluation (free energy and hybridization probability) of “a”/“a*” crosslinker units in EcoRI-responsive **MD1**

(Panel I), and “c”/“c*” crosslinker units in a second HindIII-responsive **MD2**, by NUPACK software. (B) Panel I-Temporal average size changes corresponding to the temporal growth of **MD1** in the presence of 30 μM “a”/“a*” (10 μM **Y1** and 15 μM (**4**)/(**5**)), or in the presence of 18 μM “a”/“a*” (6 μM **Y1** and 9 μM (**4**)/(**5**)). Panel II-Temporal average size changes corresponding to the temporal growth of **MD2** in the presence of 18 μM “c”/“c*” (6 μM **Y2** and 9 μM (**9**)/(**10**)) (C) Temporal confocal fluorescence microscopy images (scale bar = 50 μm) of **MD1** in the presence of 18 μM “a”/“a*” (6 μM **Y1** and 9 μM (**4**)/(**5**)).

It should be noted that the sequences of the duplex fuel domains guiding the evolution of the endonuclease formation of the endonuclease-responsive MDs shown in Figure 1 (EcoRI-responsive) and Figure S2 (HindIII-responsive) are important parameters controlling the rate and size of the evolved MDs. In fact, by computational evaluation of the thermodynamic stabilities (free energy) of the resulting duplexes between the “a”-terminated **Y1** and “a*”-terminated (**4**)/(**5**) duplex, and the stability of “c”-terminated **Y2** and “c*”-terminated (**9**)/(**10**) duplex using the NUPACK software, one may predict the probability of duplex formation, and thus the anticipated rates of MD formation. This is presented in Figure S5(A), demonstrating that the free energy of forming the “a”/“a*” duplex is -9.73 kcal/mol, Panel I, and of the “c”/“c*” duplex is -9.90 kcal/mol, Panel II, suggesting duplex formation probabilities of 28.5% for “a”/“a*” and 40% for “c”/“c*”. This is reflected by the fact that in the presence of the same concentration of “a”/“a*” and “c”/“c*” (18 μM), the rate of formation of the “a”/“a*”-crosslinked **MD1** (experimental results shown in Figure S5(C)) is substantially slower as compared to the rate evolving the “c”/“c*”-crosslinked **MD2**, Figure S5(B), Panel I vs. Panel II. These results suggest that to obtain comparable evolution rates of **MD1** and **MD2**, one should increase the crosslinking probability by increasing the concentration of “a”/“a*”. Indeed, increasing the concentration of “a”/“a*” from 18 μM

to 30 μM yields comparable evolution rates between **MD1** and **MD2**, Figure S5(B), Panel I.

These results demonstrate the ability to tune the rate of evolution by the fuel sequences of endonuclease-responsive MDs.

Tunable transient operation of the Non-Gated or inhibitor-Gated MD1/MD2 coacervates mixtures

The results depicted in Figure 2 demonstrate the inhibitor-guided programmed gated operation of the MD coacervates **MD1** (green) and **MD2** (red). While the gated temporal evolution/depletion of the two MDs proceeded similar time scales, the temporal gated process can be tuned by controlling the concentrations of the enzymes participatingin the gating processes. This is supported by the results presented in Figure S6 ~ Figure S8. While increasing the concentration of the two endonucleases EcoRI and HindIII to 12 U/μL lowers the peak sizes of the evolved MDs and enhances their depletion, Figure S6(A), decreasing the concentration of the endonucleases results in higher MD peak sizes and prolonged depletion time intervals, Figure S6(B). Similarly, in the presence of inhibitor (**11**), increasing the concentration of the endonucleases leads to smaller peak sizes of **MD1** and enhanced depletion rates, Figure S7(A), while decreasing the concentration of the endonucleases results in larger peak sizes of **MD1** and prolonged depletion time intervals, Figure S7(B). Alternatively, in the presence of inhibitor (**12**), increasing the concentration of the endonucleases leads to smaller peak sizes of **MD2** and enhanced depletion rates, Figure S8(A), while decreasing the concentration of the endonucleases results in larger peak sizes of **MD2** and prolonged depletion time intervals, Figure S8(B).

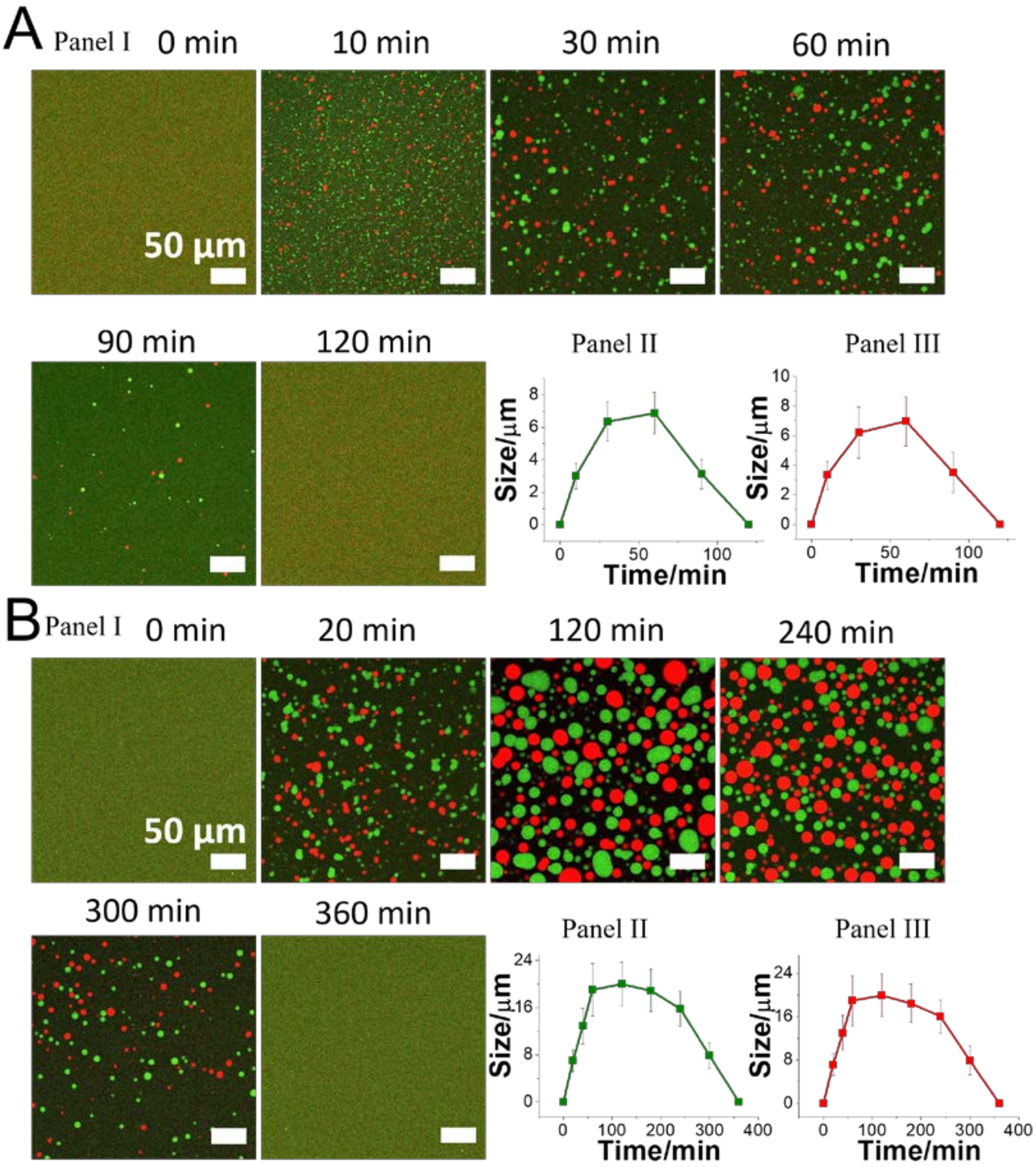

Figure S6. (A) Temporal confocal fluorescence microscopy images (scale bar = 50 μm) (Panel I) and temporal average size changes of **MD1** (Panel II)and **MD2** (Panel III) of Non-gated MDs assembly operating the dual formation and depletion of the transient EcoRI- and HindIII-responsive MDs (**MD1** and **MD2**), in the presence of 12 U/μL EcoRI and HindIII. (B) Temporal confocal fluorescence microscopy images (scale bar = 50 μm) (Panel I) and temporal average size changes of **MD1** (Panel II)and **MD2** (Panel III) of Non-gated MDs assembly operating the dual formation and depletion of the transient EcoRI- and HindIII-responsive MDs (**MD1** and **MD2**), in the presence of 4 U/μL EcoRI and HindIII.

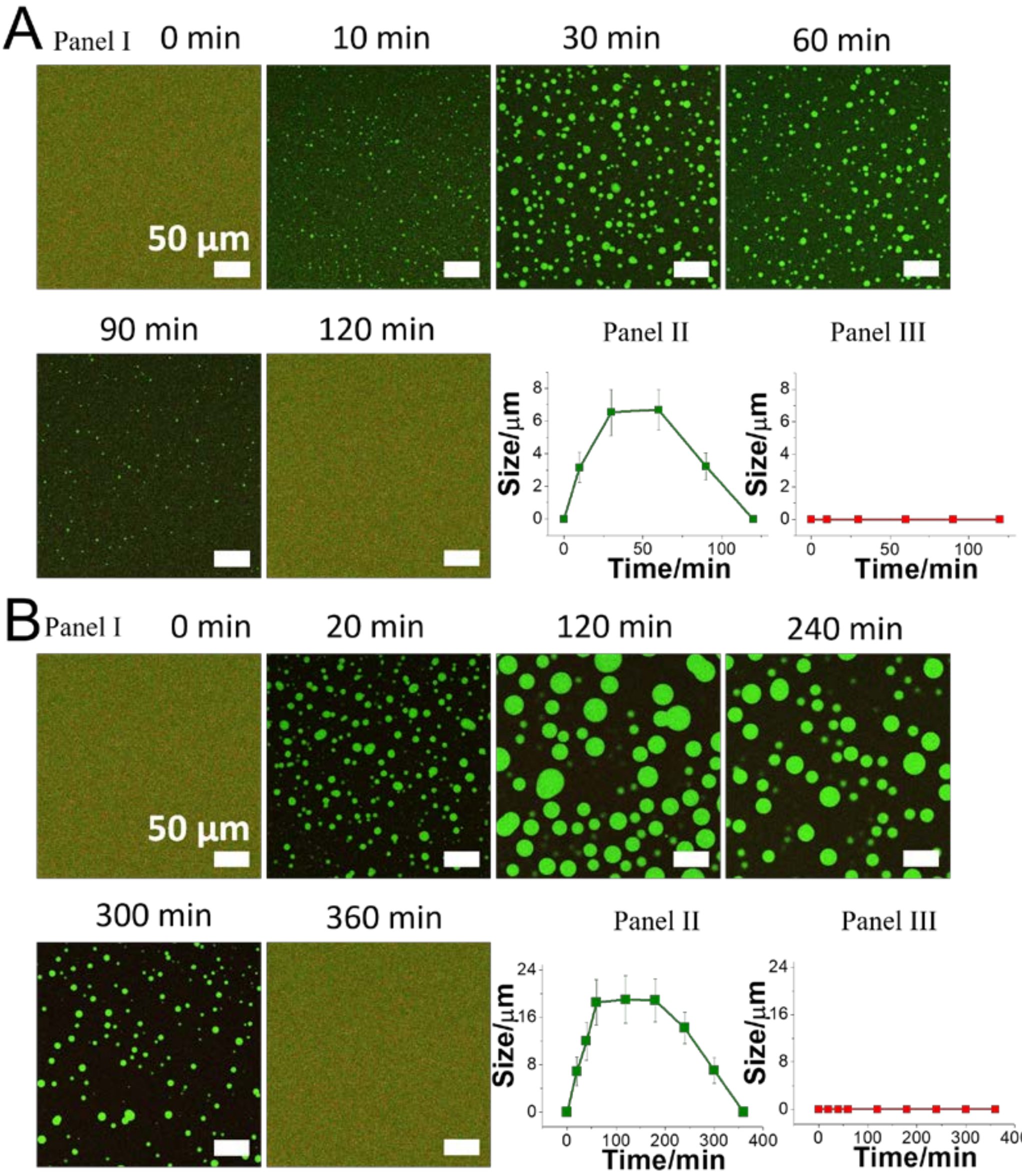

Figure S7. (A) Temporal confocal fluorescence microscopy images (scale bar = 50 μm) (Panel I) and temporal average size changes of **MD1** (Panel II)and **MD2** (Panel III) of Gated MDs assembly operating inhibitor (**11**)-gated blockage of HindIII-responsive MDs (**MD2**) and transient evolution/depletion of EcoRI-responsive MDs (**MD1**), in the presence of 12 U/μL EcoRI and HindIII. (B) Temporal confocal fluorescence microscopy images (scale bar = 50 μm) (Panel I) and temporal average size changes of **MD1** (Panel II)and **MD2** (Panel III) of Gated MDs assembly operating inhibitor (**11**)-gated blockage of HindIII-responsive MDs (**MD2**) and transient evolution/depletion of EcoRI-responsive MDs (**MD1**), in the presence of 4 U/μL EcoRI and HindIII.

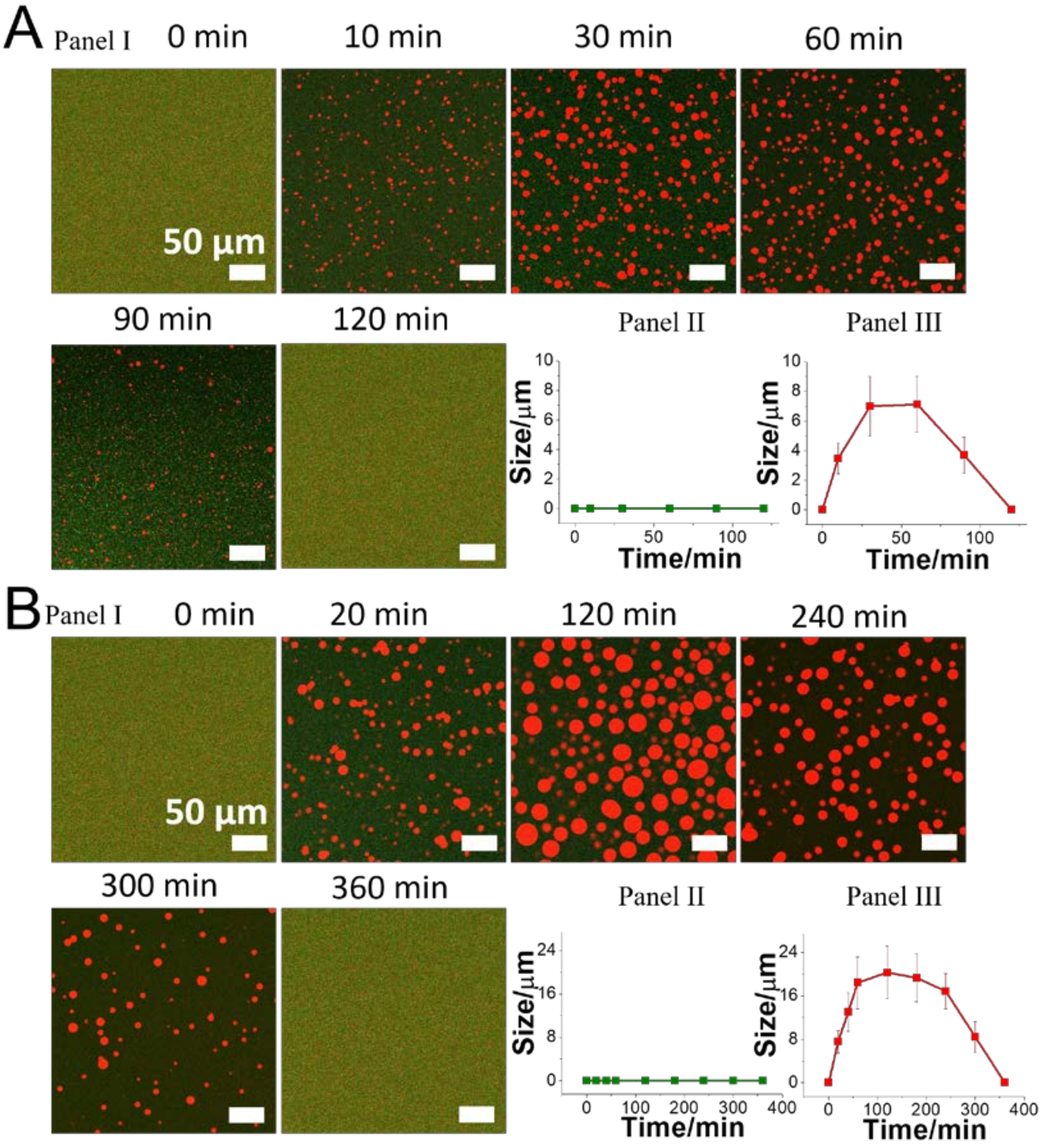

Figure S8. (A) Temporal confocal fluorescence microscopy images (scale bar = 50 μm) (Panel I) and temporal average size changes of **MD1** (Panel II) and **MD2** (Panel III) of Gated MDs assembly operating inhibitor (**12**)-gated blockage of EcoRI-responsive MDs (**MD1**) and transient evolution/depletion of HindIII-responsive MDs (**MD2**), in the presence of 12 U/μL EcoRI and HindIII. (B) Temporal confocal fluorescence microscopy images (scale bar = 50 μm) (Panel I) and temporal average size changes of **MD1** (Panel II)and **MD2** (Panel III) of Gated MDs assembly operating inhibitor (**12**)-gated blockage of EcoRI-responsive MDs (**MD1**) and transient evolution/depletion of HindIII-responsive MDs (**MD2**), in the presence of 4 U/μL EcoRI and HindIII.

Specificity of MD1 and MD2 in the presence of a foreign endonuclease

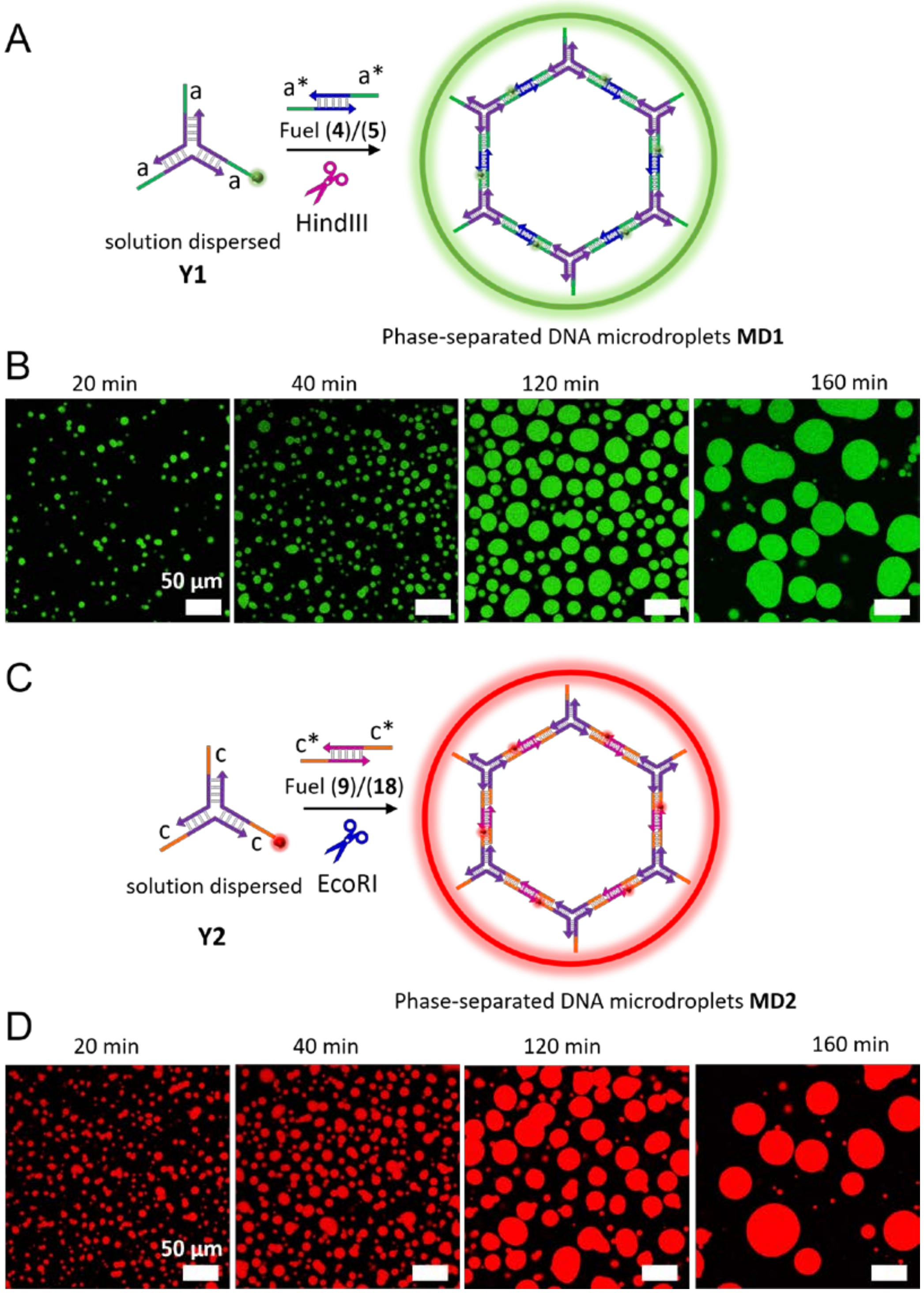

Figure S9. (A) Schematic assembly and (B) temporal confocal fluorescence microscopy images (scale bar = 50 µm) of phase-separated **MD1** by the crosslinking of Y-shaped module **Y1** with fuel (**4**)/(**5**) in the presence of a foreign HindIII endonuclease. (C) Schematic assembly and (D) temporal confocal fluorescence microscopy images (scale bar = 50 µm) of phase-separated **MD2** by the crosslinking of Y-shaped module **Y2** with fuel (**9**)/(**10**) in the presence of a foreign EcoRI endonuclease.

The specificity of EcoRI-responsive phase-separated **MD1** and HindIII-responsive phase separated **MD2** was then investigated by the assembly of MDs in the presence of a foreign endonuclease. As shown in Figure S9(A), subjecting the fuel (**4**)/(**5**) to a mixture of Y-shaped module **Y1** and HindIII endonuclease, led to the a/a* crosslinked hexagonal framework of **MD1**. While the fuel (**4**)/(**5**) was pre-engineered to be recognized and cleaved by EcoRI endonuclease, HindIII endonuclease has a different recognition sequence, which does not exist in the fuel (**4**)/(**5**). Thus, HindIII endonuclease could not digest the EcoRI-responsive **MD1**. Figure S9(B) depicted the temporal confocal fluorescence images of EcoRI-responsive **MD1** in the presence of HindIII. Within a time interval of 160 min, the phase-separated **MD1** revealed an similar average size of ca. 30 μm, as compared to the sizes of **MD1** without HindIII in Figure 1(B), which demonstrated that HindIII had almost no effect on the growth of phase-separated **MD1**. Similarly, as shown in Figure S9(C), subjecting the fuel (**9**)/(**10**) to a mixture of Y-shaped module **Y2** and EcoRI endonuclease, led to the c/c* crosslinked hexagonal framework of **MD1**. While the fuel (**9**)/(**10**) was pre-engineered to be recognized and cleaved by HindIII endonuclease, EcoRI endonuclease has a different recognition sequence, which is lack in the fuel (**9**)/(**10**). Accordingly, EcoRI endonuclease could not digest the HindIII-responsive **MD2**. Figure S9(D) depicted the temporal confocal fluorescence images of **MD2** in the presence of EcoRI. Within a time interval of 160 min, the phase-separated **MD2** revealed an similar average size of ca. 30 μm, as compared to the sizes of **MD2** without EcoRI in Figure S2(B), indicating that EcoRI had almost no effect on the growth of phase-separated **MD2**.

Orthogonal formation of phase-separated MD1 and MD2

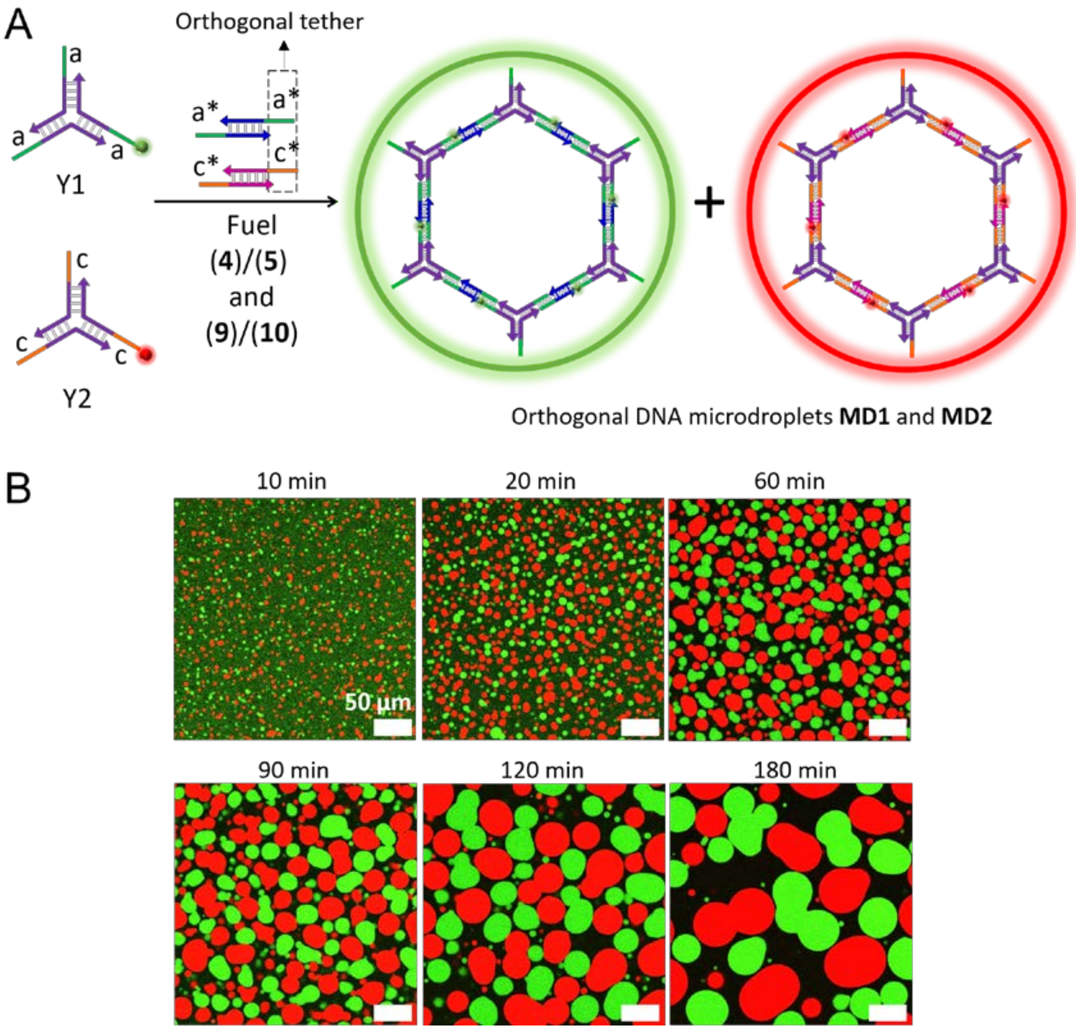

Figure S10. (A) Schematic assembly and (B) temporal confocal fluorescence microscopy images (scale bar = 50 μm) of orthogonal phase-separated MDs **MD1** and **MD2** using a mixture of Y-shaped module **Y1** and **Y2**, and a mixture of fuel (**4**)/(**5**) and (**9**)/(**10**).

Two kinds of fluorophore-labeled phase-separated MDs **MD1** and **MD2** was constructed separately, which inspired us to further develop the complexed MDs system exhibiting orthogonal transient MDs system, and subsequently inhibitors-gated transient MDs system, or selective transient MDs system. The schematic assembly of two orthogonal MDs **MD1** and **MD2** was depicted in Figure S10. A mixture of fuel (**4**)/(**5**) and (**9**)/(**10**) was subjected to the mixture of Y-shaped module **Y1** and **Y2**,

leading to the a/a* and c/c* crosslinked framework. As the crosslinked toehold a/a* and c/c* were engineered to be orthogonal, there is no interaction between two crosslinked frameworks, leading to separate, orthogonal phase-separated MDs **MD1** (green fluorescence)and **MD2** (red fluorescence). Figure S10(B) displayed the temporal confocal fluorescence microscopy images, demonstrating the dynamic growth of two orthogonal MDs. Two separate, independent, orthogonal fluorescent MDs **MD1** exhibiting green fluorescence and **MD2** exhibiting red fluorescence were observed during the growth of MDs, both of which reached the average size of ca. 30 μm within the time interval of 150 min.

Assembly/depletion of nickase (Nt.BbvCI)-responsive phase-separated microdroplets MD3

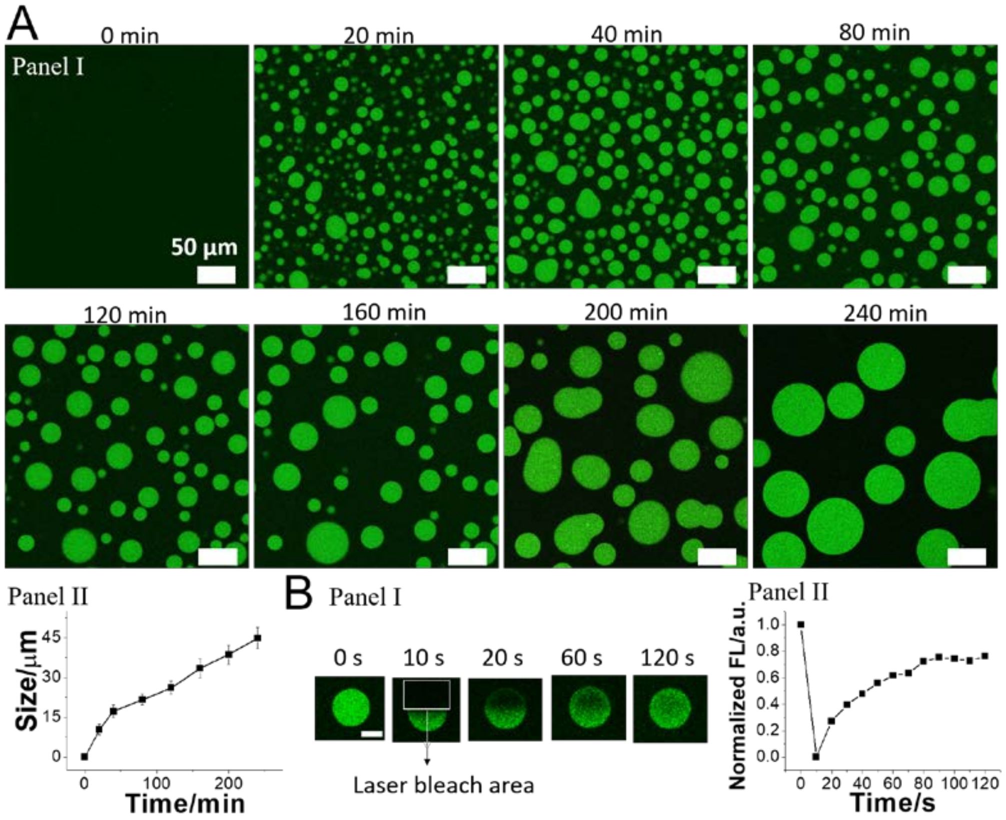

Figure S11. (A) Panel I-Temporal confocal fluorescence microscopy images (scale bar = 50 μm) of phase-separated microdroplets **MD3**, composed of strand (**16**) fueled crosslinked **Y3** framework. Panel II-Average size changes corresponding to the temporal growth of **MD3**. (C) Panel I-Temporal confocal fluorescence microscopy images (scale bar = 5 μm) of a single **MD3** corresponding to the fluorescence recovery after photobleaching (FRAP) in the upper domain of a single **MD3**. Panel II-Temporal normalized fluorescence intensity changes in the FRAP domain of the single microdroplet **MD3**.

Subjecting the palindromic domain- tethered fuel strand (**16**) to the green fluorophore-labeled Y-shaped module **Y3** generated the green fluorescent phase-separated **MD3**. Figure S11(A), Panel I displayed the temporal confocal fluorescence microscopy images of phase-separated microdroplets **MD3**, revealing dynamic growth features. The temporal average size of **MD3** was depicted in Figure S11(A), Panel II,

reaching ca. 45 μm within a time interval of 240 min. The liquid-like features of **MD3** was investigated by FRAP experiment in Figure S11(B). As shown in Figure S11(B) Panel I, the upper domain of a single **MD3** was chosen as the bleach region of FRAP, where an intensified confined laser was applied for 2 s at t = 10 s, leading to a totally FAM-bleached DNA constituents in the upper domain. Afterwards, the single MD was imaged at time intervals to observe the fluorescence recovery process. At t = 120 s, the upper bleached domain is almost recovered, where the recovered fluorescence originated from the non-bleached Y-shaped DNA module **Y3** from the lower domain of the MD, demonstrating the fluid, dynamically exchangeable properties of the Y-shaped DNA module in **MD3**. Figure S11(B), Panel II showed the temporal normalized fluorescence intensities in the upper bleached domain of the single **MD3**, the short recovery time of ca. 120 s indicated the fluid-like properties in the **MD3** containment, instead of gel-like properties.

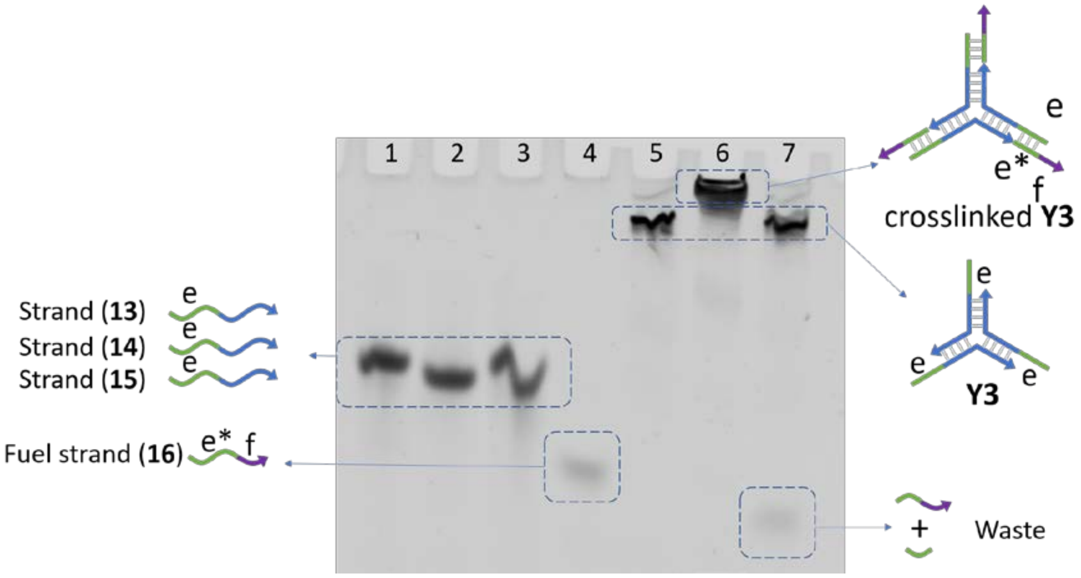

Figure S12. Gel electrophoresis analysis of the assembly of nickase Nt.BbvCI-responsive **MD3**. Lane 1: strand (**13**); lane 2: strand (**14**); lane 3: strand (**15**); lane 4: strand (**16**); lane 5: **Y3** = (**13**)/(**14**)/(**15**); lane 6: **Y3** + Fuel (**16**); lane 7: **Y3** + Fuel (**16**) + HindIII.

The assembly/depletion of nickase Nt.BbvCI-controlled microdroplets **MD3** was characterized by gel electrophoresis experiment, Figure S12. The assembly of Y-shaped module **Y3** was confirmed in the band of lane 5, as compared to the band of component strands in lane 1 ~ lane 3. While the incubation of the Y-shaped module **Y3** with the palindromic domain tethered fuel (**16**) led to the crosslinked framework (lane 6) in the **MD3**, subjecting the Nt.BbvCI-cleavable **MD3** framework to Nt.BbvCI led to the digestion of the crosslinked framework, recovering the band the Y-shaped module **Y3** and generating the band of waste (lane 7).

Transient assembly/depletion of nickase (Nb.BtsI)-responsive phase-separated microdroplets MD4

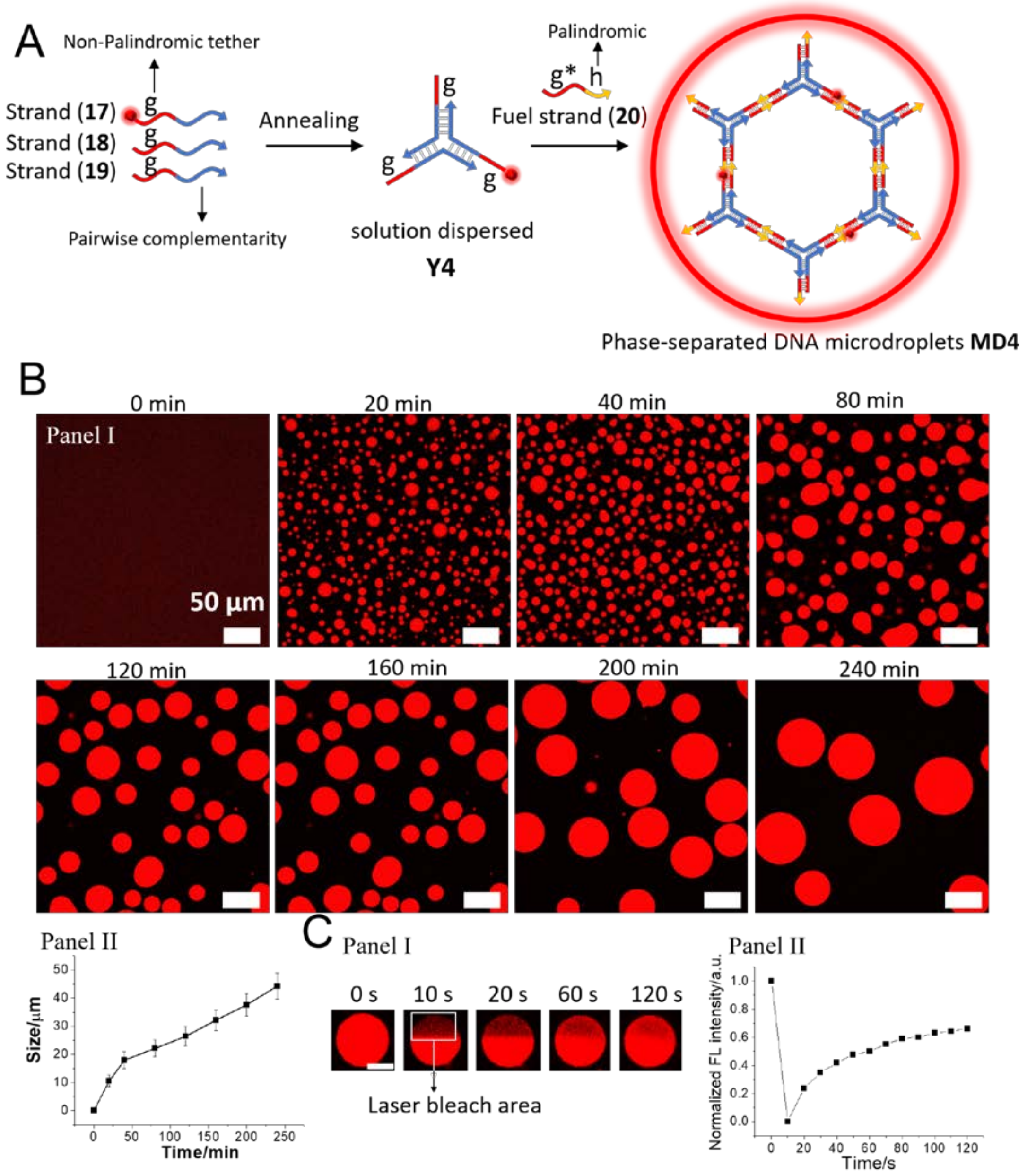

Figure S13. (A) Schematic assembly of a second nickase Nb.BtsI-responsive **MD4** by the crosslinking of Cy5-labeled Y-shaped module **Y4** with a fuel strand (**20**) with palindromic tether. (B) Panel I-Temporal confocal fluorescence microscopy images (scale bar = 50 μm) of phase-separated microdroplets **MD4**. Panel II-Average size changes corresponding to the temporal growth of **MD4**. (C) Panel I-Temporal confocal fluorescence microscopy images (scale bar = 5 μm) of a single **MD4** corresponding to the fluorescence recovery after photobleaching (FRAP) in the upper domain of the **MD4**. Panel II-Temporal normalized fluorescence intensity changes in the FRAP domain of the single **MD4**.

The nicakse-dictated cleavage of specific base sequence in DNA duplex was, then applied to design a second type of Nb.BtsI-driven transient assembly/depletion of Cy5-labeled MDs **MD4**, Figures S13 ~ S15. The assembly of the MDs **MD4** was depicted in Figure S13(A). The annealing of a mixture of components (**17**)/(**18**)/(**19**) generated the Y-shaped module **Y4** with arm-extended free toehold tethers g. (Note that 10% of strand (**17**) was labeled with a red-fluorescent Cy5 fluorophore.) The fuel strand (**20**) was engineered to include g-complementary domain g*, extended by a palindromic (self-complementary) tether h. Accordingly, subjecting appropriate amount of fuel (**20**) to Y-shaped module **Y4** generated g/g* hybridized (**20**)/**Y4** Y-shaped module with h tethers, leading to the self-crosslinking of Y-shaped module (**20**)/**Y4** and the subsequent red-fluorescent phase-separated DNA microdroplets **MD4**. Figure S13(B), Panel I displayed the temporal confocal fluorescence microscopy images of phase-separated MDs **MD4**, revealing dynamic growth features. The statistical average sizes of **MD4** was depicted in Figure S13(B), Panel II. The phase-separated MDs **MD4** revealed an average size distribution of ca. 45 μm within a time interval of 240 min. The fluidic properties of **MD4** was then investigated by FRAP experiment, Figure S13(C). As shown in Figure S13(C), Panel I, the upper domain of a single **MD4** was chosen as the bleached domain of FRAP, where an intensified confined laser was applied for 2 s at t = 10 s, resulting in a totally Cy5-bleached DNA constituents in the upper domain. Afterwards, the single MD was imaged at time intervals to observe the fluorescence recovery process. At t = 120 s, the upper bleached domain is almost recovered, where the recovered fluorescence originated from the non-bleached DNA Y-shaped module

Y4 from the lower domain of the MD, demonstrating the fluid, dynamically exchangeable properties of the DNA Y-shaped module in **MD4**. Figure S13(C), Panel II showed the temporal normalized fluorescence intensities in the upper bleached domain of the single MD, the short recovery time of ca. 120 s indicated the fluid-like properties in the **MD4** containment, instead of gel-like properties.

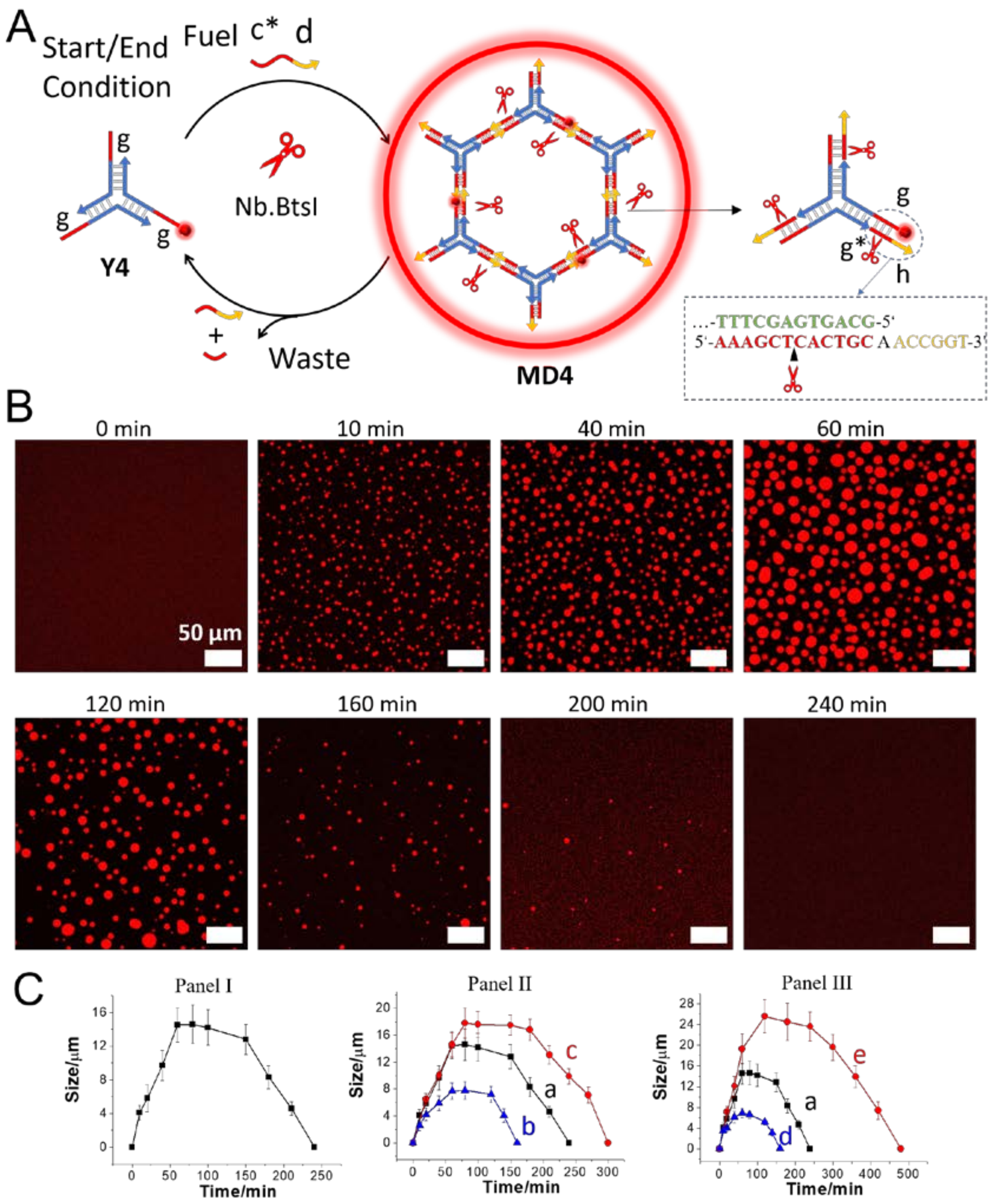

Figure S14. (A) Schematic transient formation/depletion of a second nickase Nb.BtsI -controlled microdroplets **MD4** by the fueled crosslinking of Cy5-labeled **Y4** with a

fuel strand (**20**) in the presence of Nb.BtsI. (B) Temporal confocal fluorescence microscopy images (scale bar = 50 μm) of transient dissipative formation/depletion of phase-separated microdroplets **MD4**. (C) Panel I-Temporal average size changes of phase-separated **MD4**, in the presence of **Y4**, 10 μM, fuel (**20**), 30 μM, and Nb.BtsI, 1 U/μL. Panel II-Temporal average size changes of phase-separated **MD4**, in the presence of **Y4**, 10 μM, and Nb.BtsI, 1 U/μL, and variable concentrations of fuel (**20**): (a) 30 μM, (b) 24 μM, (c) 36 μM. Panel III-Temporal average size changes of phase-separated **MD4**, in the presence of **Y4**, 10 μM, fuel (**20**), 30 μM, and variable concentrations of Nb.BtsI, (a) 1 U/μL, (d) 1.5 U/μL, (e) 0.5 U/μL.

The Nb.BtsI-dictated transient, dissipative formation and depletion of phase-separated MDs **MD4** was schematically displayed in Figure S14(A). Subjecting the fuel (**20**) to a mixture of Y-shaped module **Y4** and Nb.BtsI nickase, generated the self-crosslinking of hybridized (**20**)/**Y4** units, resulting in the phase-separated **MD4**. The fuel (**20**) was, however, pre-engineered to be specifically recognized and cleaved by Nb.BtsI nickase upon hybridization, leading to the concomitant cleavage of the fuel (**20**) and self-crosslinked framework of **MD4**. The concomitant digestion of **MD4** limited the growth of phase-separated **MD4**, and ultimately depleted all the MDs, regenerating the solution-dispersed Y-shaped module **Y4** with the cleaved waste products. The transient time-dependent growth and depletion of the **MD4** was probed by the confocal fluorescence microscopy images, Figure S14(B). Within ca. 60 min, the growth of the **MD2** proceeded, reaching a maximum average size of 14 μm. The **MD4** started to be degraded afterwards, with the less content of MDs and decreased average sizes, which was fully depleted after 240 min. The temporal size changes of the **MD4** was depicted in Figure S14(C) Panel I, and the sizes and transient depletion time of **MD4** are controlled by the concentration of fuel (**20**) and the concentration of Nb.BtsI, Figure S14(C) Panel II, and Panel III, respectively. As the concentration of fuel (**20**) increases,

the maximum size of **MD4** is larger and the total depletion time is longer. As the concentration of Nb.BtsI increases, the peak size of **MD4** is smaller with a shorter transient depletion time.

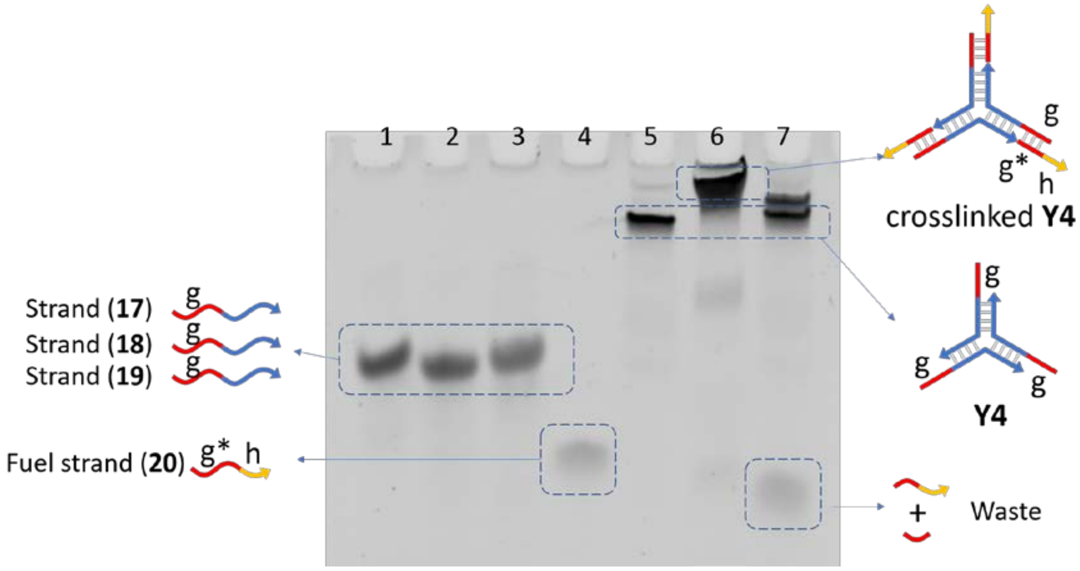

Figure S15. Gel electrophoresis analysis of the assembly of nickase Nb.BtsI-controlled **MD4**. Lane 1: strand (**17**); lane 2: strand (**18**); lane 3: strand (**19**); lane 4: strand (**20**); lane 5: **Y4** = (**17**)/(**18**)/(**19**); lane 6: **Y3** + Fuel (**20**); lane 7: **Y4** + Fuel (**20**) + Nb.BtsI.

The assembly/depletion of Nb.BtsI-controlled microdroplets **MD4** was analyzed by gel electrophoretic experiment, Figure S15. The assembly of Y-shaped module **Y4** was evidenced in the band of lane 5, as compared to the band of constitutional strands in lane 1 ~ lane 3. While the incubation of the Y-shaped module **Y4** with the fuel (**20**) led to the self-crosslinked framework (lane 6) in the **MD4**, the Nb.BtsI-responsive **MD4** framework was depleted by Nb.BtsI digestion, recovering the band the Y-shaped module **Y4** and generating the band of waste (lane 7).

Specificity of MD3 and MD4 in the presence of a foreign nickase

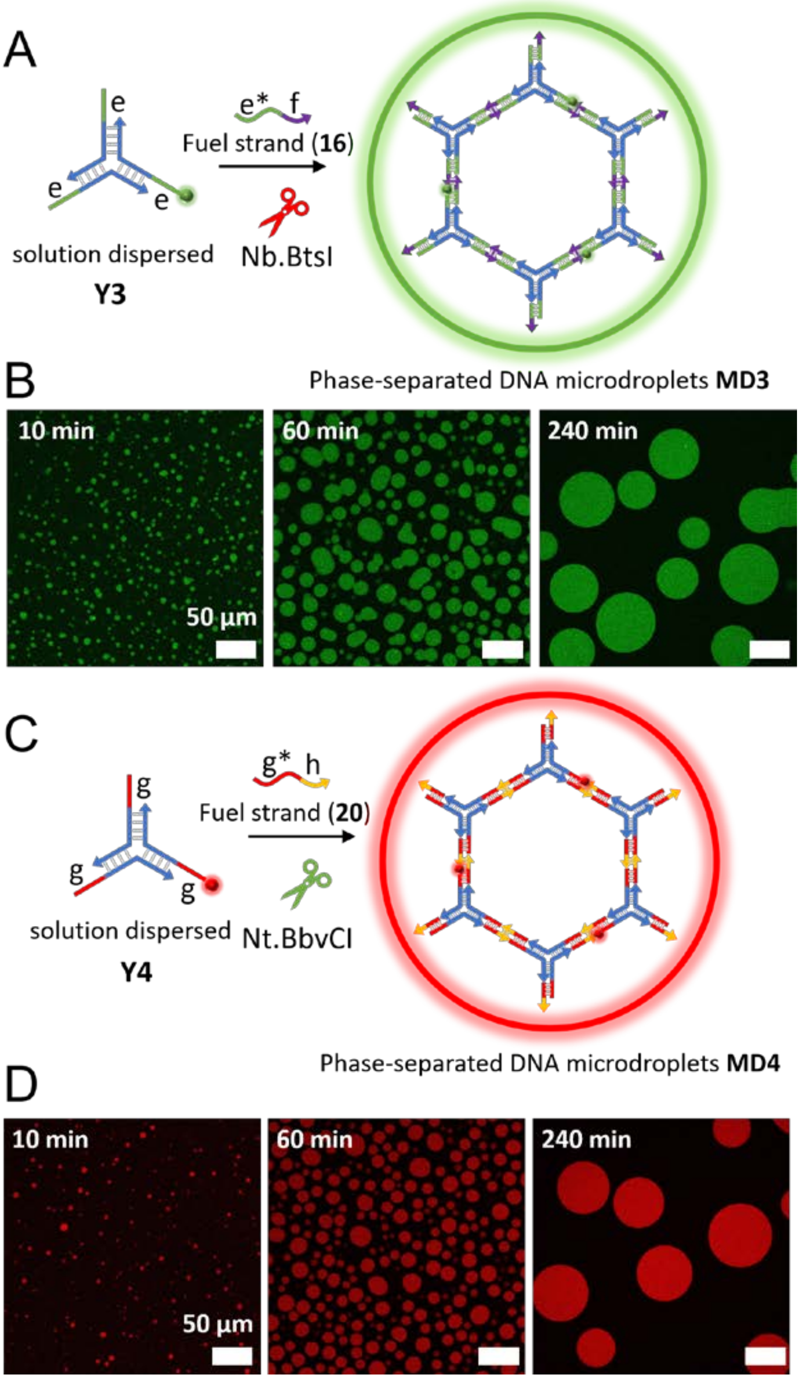

Figure S16. (A) Schematic assembly and (B) temporal confocal fluorescence microscopy images (scale bar = 50 μm) of phase-separated **MD3** by the subjection of Y-shaped module **Y3** with fuel (**16**) in the presence of a foreign Nb.BtsI nickase. (C) Schematic assembly and (D) temporal confocal fluorescence microscopy images (scale bar = 50 μm) of phase-separated **MD4** by the incubation of Y-shaped module **Y4** with fuel (**20**) in the presence of a foreign Nt.BbvCI nicakse.

The specificity of Nt.BbvCI-responsive phase-separated **MD3** and Nb.BtsI-

responsive phase separated **MD4** was then investigated by the assembly of MDs in the presence of a foreign nickase. As shown in Figure S16(A), subjecting the fuel (**16**) to Y-shaped module **Y3** in the presence of Nb.BtsI nickase, led to the self-crosslinked hexagonal framework of **MD3**. While the fuel (**16**) was pre-engineered to be recognized and cleaved by Nt.BbvCI, Nb.BtsI nickase has a different recognition sequence, which does not exist in the fuel (**16**). Thus, Nb.BtsI could not digest the Nt.BbvCI-responsive **MD3**. Figure S16(B) depicted the temporal confocal fluorescence images of Nt.BbvCI-responsive **MD3** in the presence of Nb.BtsI. Within a time interval of 240 min, the phase-separated **MD3** revealed an average size of ca. 45 μm, demonstrating that Nb.BtsI had no effect on the growth of phase-separated **MD3**. Similarly, as shown in Figure S16(C), subjecting the fuel (**20**) to **Y4** in the presence of Nt.BbvCI nickase, led to the self-crosslinked hexagonal framework of **MD4**. The fuel (**20**) lacks the recognition sequence of Nt.BbvCI. Accordingly, EcoRI endonuclease could not digest the Nb.BtsI-responsive **MD4**. Figure S16(D) depicted the temporal confocal fluorescence images of **MD4** in the presence of Nt.BbvCI. Within a time interval of 240 min, the phase-separated **MD4** revealed an similar average size of ca. 45 μm, indicating that Nt.BbvCI had almost no effect on the growth of phase-separated **MD4**.

Orthogonal, and selective transient formation/depletion of phase-separated MD3 and MD4

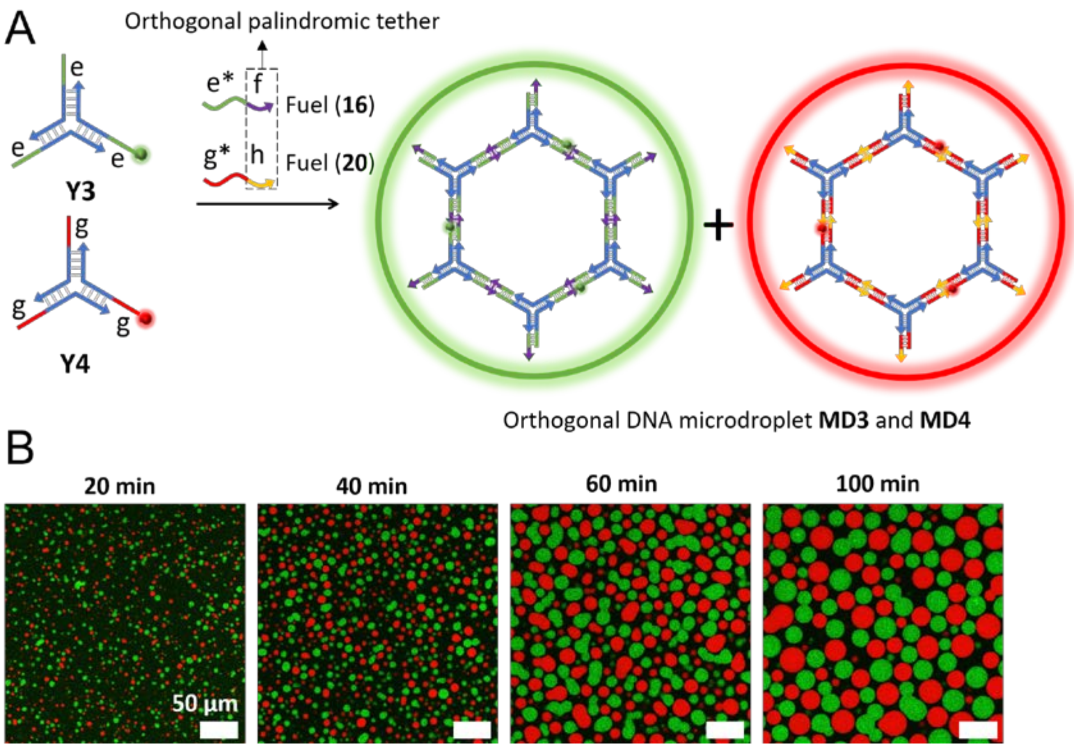

Figure S17. (A) Schematic assembly and (B) temporal confocal fluorescence microscopy images (scale bar = 50 µm) of orthogonal phase-separated MDs **MD3** and **MD4** using a mixture of Y-shaped module **Y3** and **Y4**, and a mixture of fuel (**16**) and (**20**).

The schematic assembly of two kinds of orthogonal MDs **MD3** (green fluorescence) and **MD4** (red fluorescence) was depicted in Figure S17(A). A mixture of fuel (**16**) and (**20**) was subjected to the mixture of Y-shaped module **Y3** and **Y4**, leading to the hybridized (**16**)/**Y3** module and (**20**)/**Y4** module. As the self-complementary tethers f and h associated with (**16**)/**Y3** module and (**20**)/**Y4** module were engineered to be orthogonal, there is no interaction between two self-crosslinked frameworks, leading to separate, orthogonal phase-separated MDs **MD3** (green fluorescence)and **MD4** (red fluorescence). Figure S17(B) displayed the temporal

confocal fluorescence microscopy images, demonstrating the dynamic growth of two orthogonal MDs. Two separate, independent, orthogonal fluorescent MDs **MD3** exhibiting green fluorescence and **MD4** exhibiting red fluorescence were observed, reaching similar average sizes of ca. 20 μm within the time interval of 100 min.

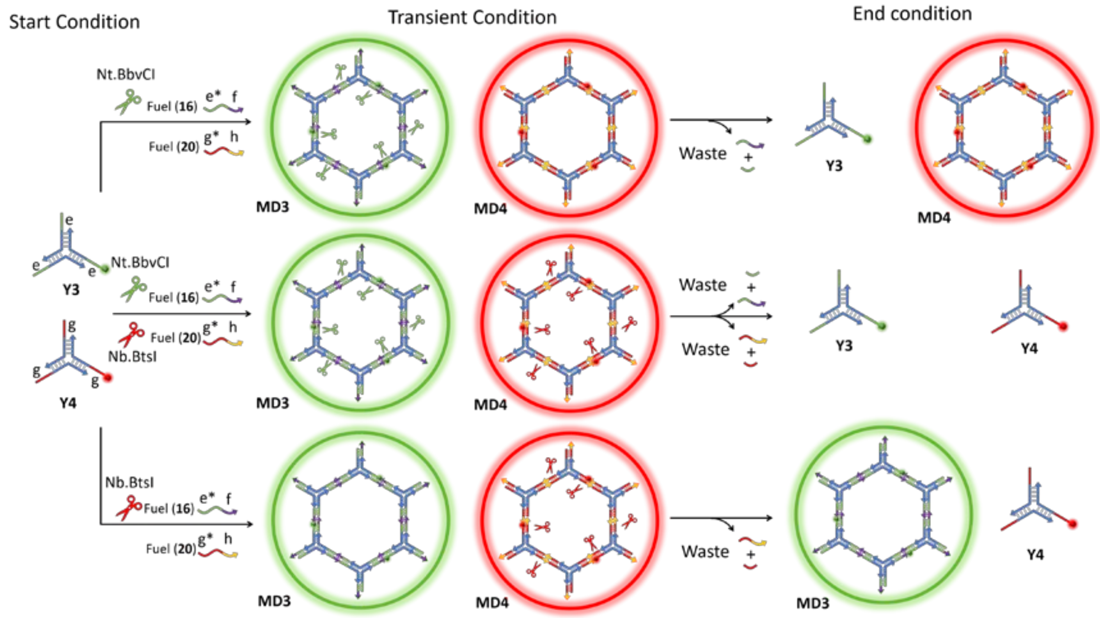

Figure S18. Schematic assembly of orthogonal or selective, transient assembly/depletion of phase-separated MDs **MD3** and **MD4** using a mixture of Y-shaped module **Y3** and **Y4**, and a mixture of fuel (**16**) and (**20**), in the presence of two nickases (Nt.BbvCI and Nb.BtsI), or a single nickase, Nt.BbvCI (B) or Nb.BtsI (C).

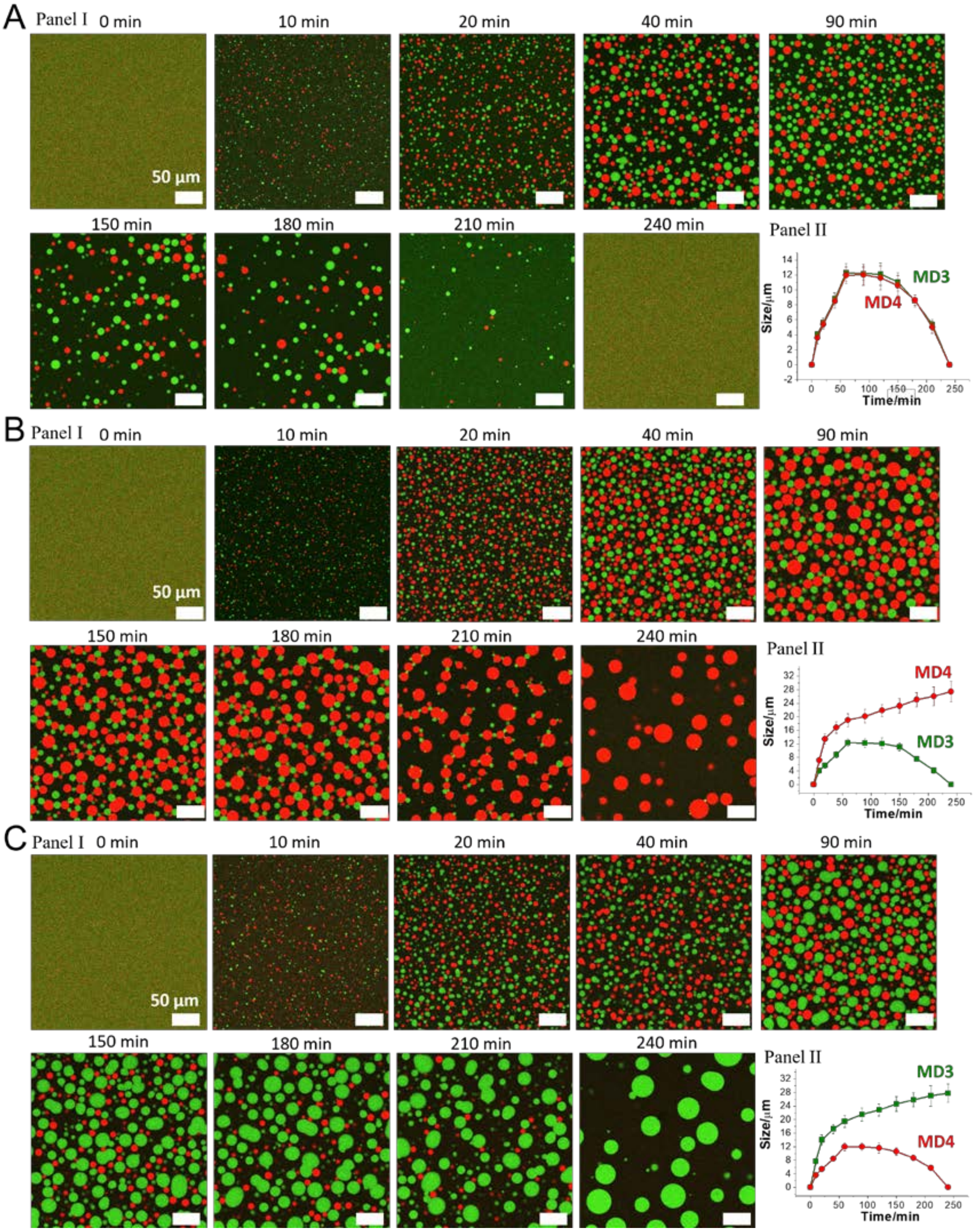

Figure S19. Temporal confocal fluorescence microscopy images (scale bar = 50 μm) of **MD3** and **MD4** (Panel I), and temporal average size changes of **MD3** and **MD4** (Panel II), corresponding to orthogonal or selective assembly/depletion of phase-separated MDs **MD3** and **MD4** using a mixture of Y-shaped module **Y3** and **Y4**, and a mixture of fuel (**16**) and (**20**), in the presence of two nickases (Nt.BbvCI and Nb.BtsI) (A), or a single nickase, Nt.BbvCI (B) or Nb.BtsI (C).

The orthogonal or selective transient MDs mixture system dictated by Nt.BbvCI and Nb.BtsI nickases was schematically shown in Figure S18. A mixture of fuel (**16**)

and (**20**) was subjected to the mixture of Y-shaped module **Y3** and **Y4** in the presence of Nt.BbvCI and Nb.BtsI, resulting in the transient formation of two orthogonal MDs **MD3** and **MD4**. As the Nt.BbvCI and Nb.BtsI selectively cleaved the fuel (**16**) and (**20**) in the framework of **MD3** and **MD4**, respectively, the **MD3** and **MD4** was transiently depleted ultimately. While subjecting the mixture of fuel (**16**) and (**20**) to the mixture of **Y3** and **Y4** in the presence of Nt.BbvCI, led to the transient assembly/depletion of **MD3** and non-inhibited growth of **MD4**, as the Nt.BbvCI selectively cleaved the fuel (**16**) in the framework of **MD3**. Alternatively, subjecting the mixture of fuel (**16**) and (**20**) to the mixture of **Y3** and **Y4** in the presence of Nb.BtsI, resulted in the transient formation and depletion of **MD4**, while **MD3** kept dynamically growing without degradation, due to the selectivity of Nb.BtsI towards **MD4**. Figure S19(A) Panel I displayed the temporal confocal fluorescence microscopy images corresponding to orthogonal transient assembly/depletion of **MD3** and **MD4** in the mixture of MDs, using a mixture of **Y3** and **Y4**, and a mixture of fuel (**16**) and (**20**), in the presence of Nt.BbvCI and Nb.BtsI. The transient formation and depletion of separate, independent green fluorescent **MD3** and red fluorescent **MD4** was observed. The temporal average size changes of **MD3** and **MD4** were depicted in Figure S19(A) Panel II, displaying dynamic growth/depletion features with peak sizes of ca. 12 μm. Figure S19(B) Panel I displayed the temporal confocal fluorescence microscopy images corresponding to selective transient assembly/depletion of **MD3** from the mixture of MDs, using a mixture of **Y3** and **Y4**, and a mixture of fuel (**16**) and (**20**), in the presence of Nt.BbvCI. The transient formation and depletion of green fluorescent **MD3** is observed, while the

red fluorescent **MD4** showed dynamic growth without inhibition. The temporal average size changes of **MD3** and **MD4** were depicted in Figure S19(B) Panel II, displaying selective Nt.BbvCI-dictated dynamic growth/depletion features of **MD3**. Alternatively, Figure S19(C) Panel I displayed the temporal confocal fluorescence microscopy images corresponding to selective assembly/depletion of **MD4** from the mixture of MDs in the presence of Nb.BtsI. The transient formation and depletion of red fluorescent **MD4** is observed, while the green fluorescent **MD3** exhibited dynamic growth without depletion. The temporal average size changes of **MD3** and **MD4** were depicted in Figure S19(B) Panel II, displaying selective Nb.BtsI-dictated dynamic growth/depletion features of **MD4**.

Evaluation of the energy input/output values associated with the different enzyme-driven dissipative microdroplet system

The evolution and depletion of the different enzymatically-driven MDs are accompanied, as required for any dissipative system a fuel guided energy input forming the reaction intermediate followed by a energy output originating from the enzyme-driven separation (degradation of the intermediate). Naturally, formation of the intermediates involves duplex formation and enzymatic cleavage accompanied by duplex separation and strand reconfiguration. The processes are controlled by the sequence-dependent stabilities of the resulting intermediates and their separated products. These values can be estimated by the NUPACK software for the different frameworks.

Figure S20 ~ Figure S24 provide the intermediate structures and accompanying free energy values, leading to the input/output energy value for the different frameworks.

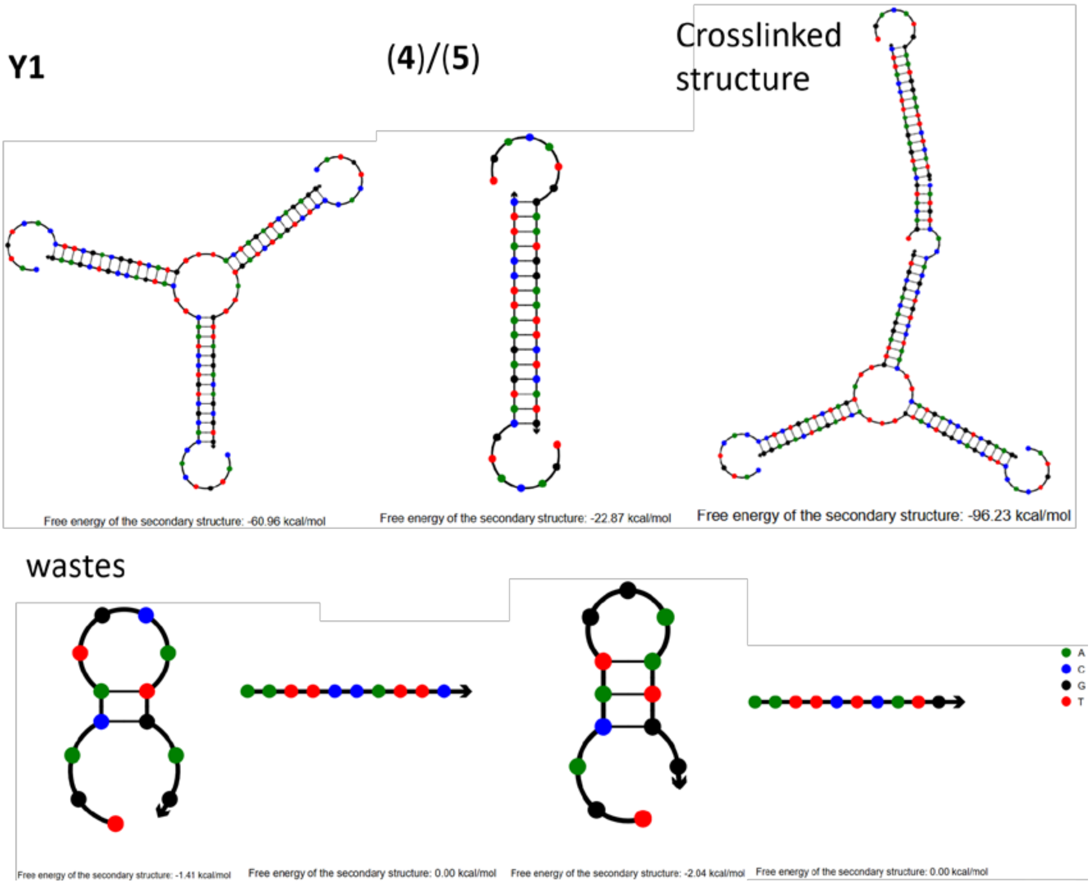

Figure S20. Assembled intermediate structures involved in the EcoRI-dictated transient formation/depletion of **MD1**.

In a typical experiment of transient formation/depletion of **MD1** (Figure 1(E), Panel I), n (**Y1**) = 0.1 nmol, n((**4**)/(**5**)) = 0.15 nmol. The intermediate hexagonal crosslinked structure has an average ratio of (**4**)/(**5**) to **Y1** to be 1.5. The computational 1:1 ratio of crosslinked (**4**)/(**5**) and **Y1** structure could not directly applied to derive the energy gain/loss of the system. ΔG(crosslinked structure 1.5:1) could be estimated to be ΔG(crosslinked structure 1:1) + (ΔG(crosslinked structure 1:1) – ΔG(Y1))/2, corresponding to a value of -113.865 kcal/mol.

ΔG(energy gain) = ΔG(**MD1**) – ΔG(**Y1**) = -5.2905 × 10^{-9} kcal = -2.215 × 10^{-5} J

ΔG(energy release) = ΔG(**Y1**) + ΔG(**waste**) – ΔG(**MD1**) = 4.773 × 10^{-9} kcal = 1.998 × 10^{-5} J

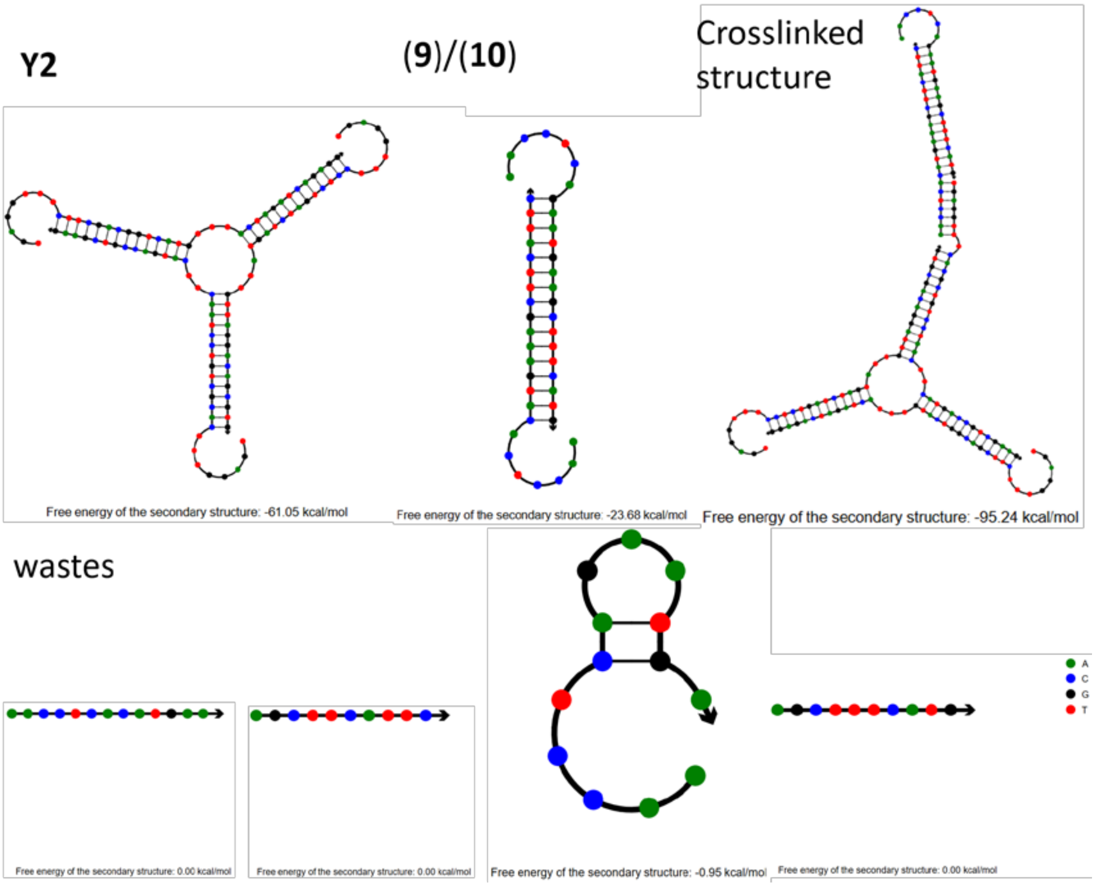

Figure S21. Computational DNA structures involved in the HindIII-dictated transient formation/depletion of **MD2**.

In a typical experiment of transient formation/depletion of **MD2** (Figure S3(B)), n (**Y2**) = 0.06 nmol, n((**4**)/(**5**)) = 0.09 nmol. The intermediate hexagonal crosslinked structure has an average ratio of (**9**)/(**10**) to **Y2** to be 1.5. The computational 1:1 ratio of crosslinked (**9**)/(**10**) and **Y2** structure could not directly applied to derive the energy gain/loss of the system. ΔG(crosslinked structure 1.5:1) could be estimated to be ΔG(crosslinked structure 1:1) + (ΔG(crosslinked structure 1:1) – ΔG(Y2))/2, corresponding to a value of -112.335 kcal/mol.

ΔG(energy gain) = ΔG(**MD2**) – ΔG(**Y2**) = -3.0771×10^{-9} kcal = -1.288×10^{-5} J

ΔG(energy release) = ΔG(**Y2**) + ΔG(**waste**) – ΔG(**MD2**) = 2.9916×10^{-9} kcal = 1.253×10^{-5} J

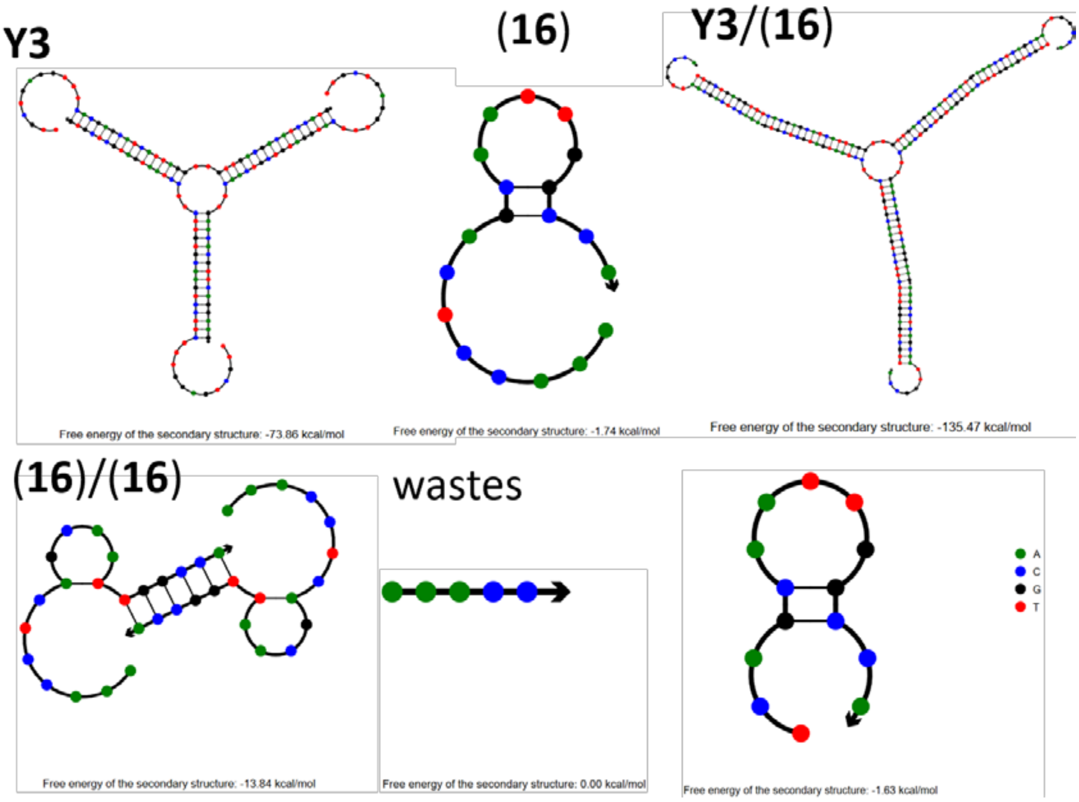

Figure S22. Computational DNA structures involved in the Nt.BbvCI-controlled transient formation/depletion of **MD3**.

In a typical experiment of transient formation/depletion of **MD3** (Figure 4(B)), n(**Y3**) = 0.1 nmol, n((**16**)) = 0.3 nmol. The intermediate hexagonal crosslinked structure has involved the stoichiometrically hybridized **Y3**/(**16**), and the subsequent self-crosslinked hybridization of (**16**)/(**16**). ΔG(crosslinker) could be estimated to be ΔG((**16**)/(**16**)) – ΔG(**16**) × 2, corresponding to a value of -10.36 kcal/mol.

ΔG(energy gain) = ΔG(**MD3**) – ΔG(**Y3**) = -7.715×10^{-9} kcal = -3.230×10^{-5} J

ΔG(energy release) = ΔG(**Y3**) + ΔG(**waste**) – ΔG(**MD3**) = 7.226×10^{-9} kcal = 3.025×10^{-5} J

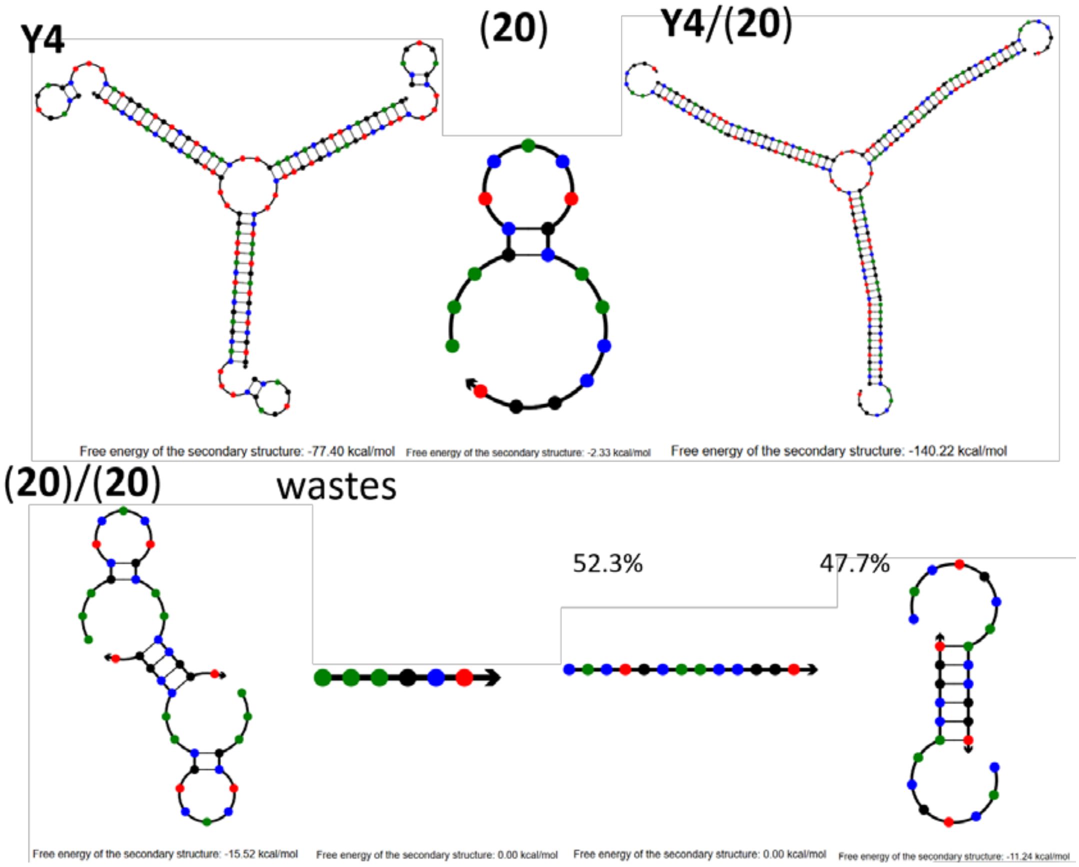

Figure S23. Computational DNA structures involved in the Nb.BtsI-controlled transient formation/depletion of **MD4**.

In a typical experiment of transient formation/depletion of **MD4** (Figure S14(B)), n (**Y4**) = 0.1 nmol, n((**20**)) = 0.3 nmol. The intermediate hexagonal crosslinked structure has involved the stoichiometrically hybridized **Y4**/(**20**), and the subsequent self-crosslinked hybridization of (**20**)/(**20**). ΔG(crosslinker) could be estimated to be ΔG((**20**)/(**20**)) – ΔG(**20**) × 2, corresponding to a value of -10.86 kcal/mol.

ΔG(energy gain) = ΔG(**MD4**) – ΔG(**Y4**) = -7.911×10^{-9} kcal = -3.312×10^{-5} J

ΔG(energy release) = ΔG(**Y4**) + ΔG(**waste**) – ΔG(**MD4**) = 7.107×10^{-9} kcal = 2.976×10^{-5} J

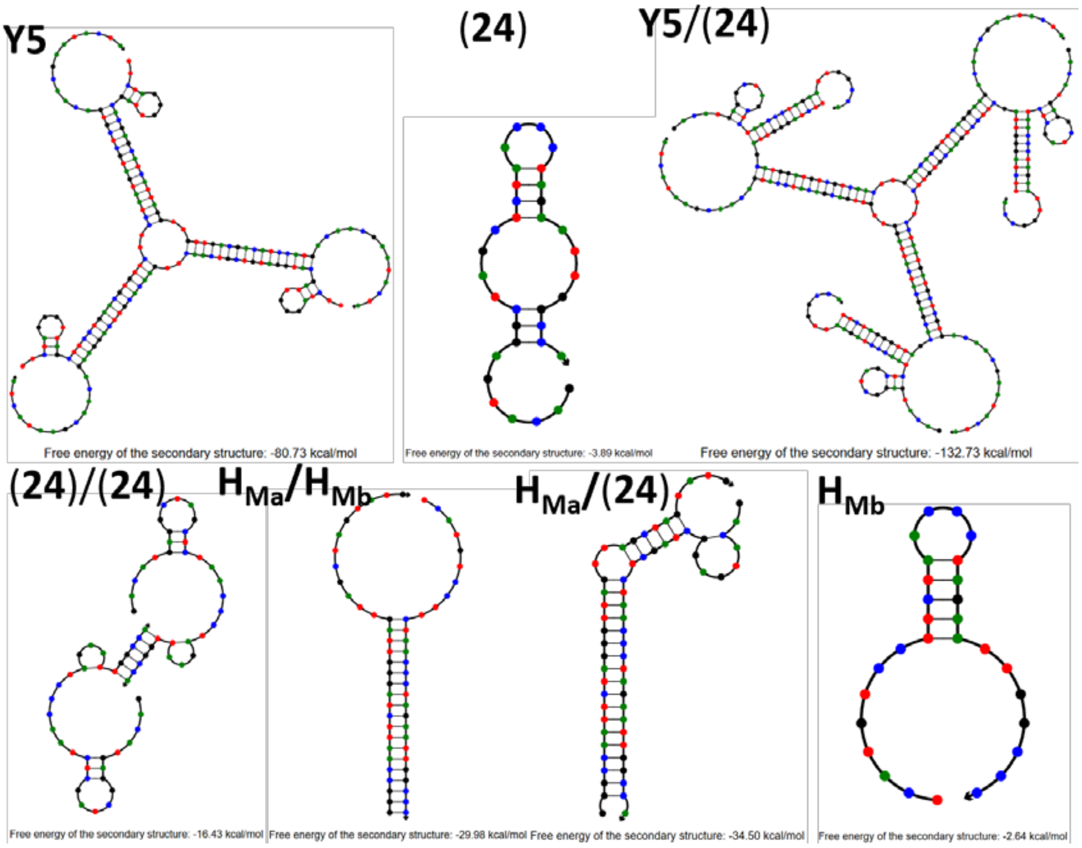

Figure S24. Computational DNA structures involved in the DNAzyme-controlled transient formation/depletion of **MD5**.

In a typical experiment of transient formation/depletion of **MD5** (Figure 5(B)), n (**Y5**) = 0.08 nmol, n((**24**)) = 0.24 nmol. The intermediate hexagonal crosslinked structure has involved the stoichiometrically hybridized **Y5**/(**24**), and the subsequent self-crosslinked hybridization of (**24**)/(**24**). ΔG(crosslinker) could be estimated to be ΔG((**24**)/(**24**)) – ΔG(**24**) × 2, corresponding to a value of -8.65 kcal/mol.

ΔG(energy gain) = ΔG(**MD5**) – ΔG(**Y5**) = -5.198×10^{-9} kcal = -2.176×10^{-5} J

ΔG(energy release) = ΔG(**Y5**) + ΔG(**waste**) – ΔG(**MD4**) – ΔG(**H_{Ma}/H_{Mb}**) = 3.480×10^{-9} kcal = 1.457×10^{-5} J

Estimation of the catalytic rates of the endonucleases/nickases in the different MDs

Knowing the concentrations of the fuel strand in the total volume of the reaction samples generating the MDs, assuming that the transient operation within the MDs consumed the entire input fuels, and knowing the experimental time intervals for depletion of the fuels, the catalytic rates of the enzymes in the different systems were estimated:

v (EcoRI) = 0.83 pmol/min. (Figure 1(E), Panel I)

v (HindIII) = 0.5 pmol/min. (Figure S3(B))

v (Nt. BbvCI) = 1.25 pmol/min. (Figure 4(B))

v (Nb. BtsI) = 1.25 pmol/min. (Figure S14(B))

Assembly/depletion of Mg^{2+}-ion-dependent DNAzyme-modified phase-separated microdroplets MD5

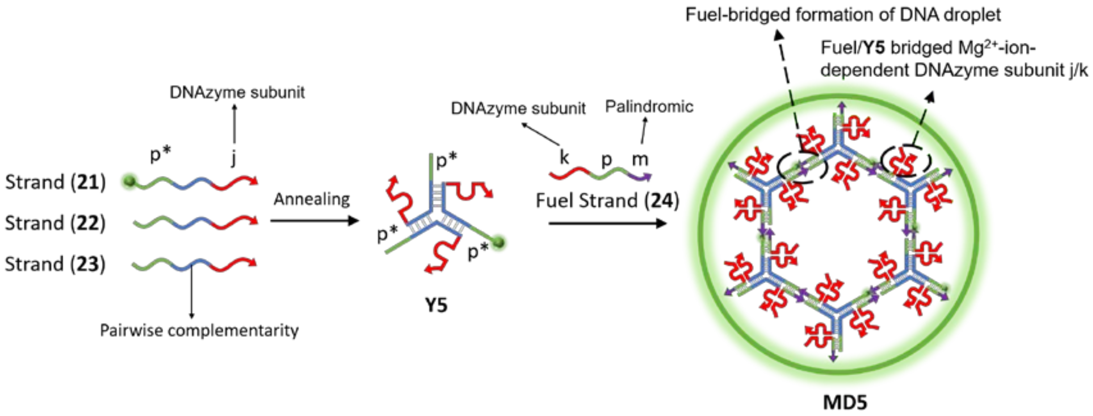

Figure S25. Schematic assembly of Mg^{2+}-ion-dependent DNAzyme-functionalized phase-separated MDs **MD5** using the Y-shaped module **Y5** and fuel strand (**24**).

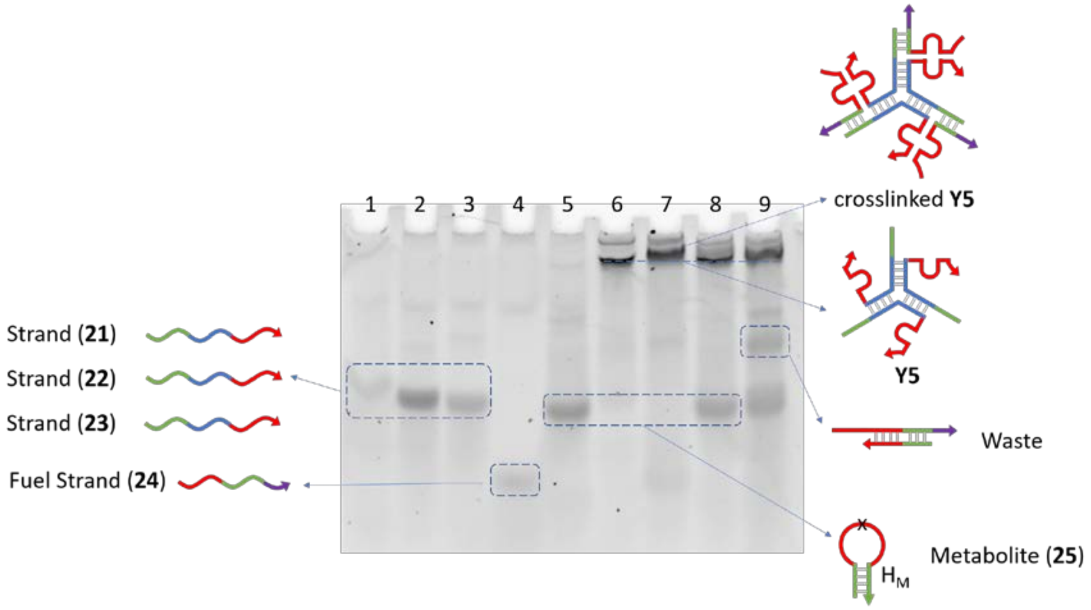

Figure S26. Gel electrophoresis analysis of the assembly of Mg^{2+}-ion-dependent DNAzyme-functionalized phase-separated MDs **MD5**. Lane 1: strand (**21**); lane 2: strand (**22**); lane 3: strand (**23**); lane 4: fuel strand (**24**); lane 5: metabolite strand (**25**); lane 6: **Y5** = (**21**)/(**22**)/(**23**); lane 7: **Y5** + Fuel (**24**); lane 8: **Y5** + metabolite (**25**); lane 9: **Y5** + Fuel (**24**) + metabolite (**25**).

The transient assembly/depletion of Mg^{2+}-ion-dependent DNAzyme-functionalized phase-separated MDs **MD5** was analyzed by gel electrophoresis experiment, Figure S26. The assembly of Y-shaped module **Y5** was observed in the

band of lane 6, as compared to the band of constitutional strands in lane 1 ~ lane 3. Subjecting the fuel (**24**) to the Y-shaped module **Y5** led to the hybridized (**24**)/**Y5**, generating self-crosslinked framework of **MD5** (lane 7). The incubation of the fuel (**24**) and **Y5** in the presence of metabolite (**25**) led to the crosslinked Mg^{2+}-ion DNAzyme framework in the **MD5**, where the metabolite (**25**) containing ribonucleobases could act as substrate of Mg^{2+}-ion DNAzyme. The cleavage of metabolite (**25**) by Mg^{2+}-ion DNAzyme generated the fragmented metabolite strand that was engineered to displace the fuel (**24**), depleting the **MD5**, recovering the band of **Y5** and generating the band of waste (lane 9).

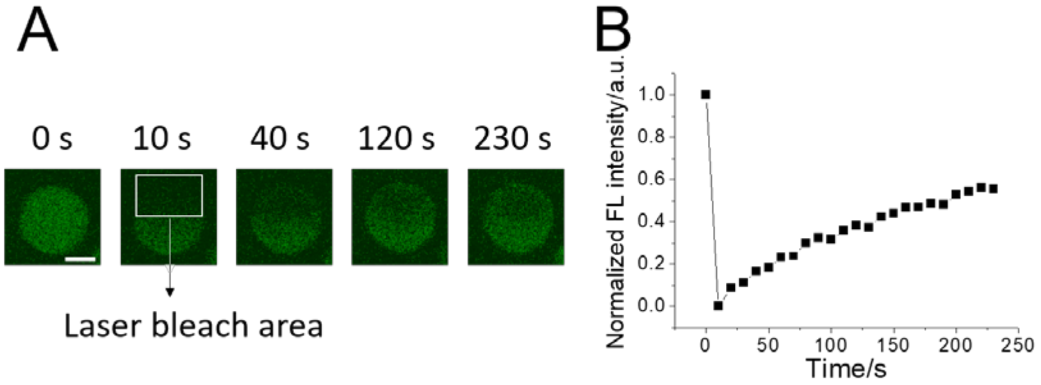

Figure S27. (A) Temporal confocal fluorescence microscopy images (scale bar = 5 μm) of a single MD **MD5** corresponding to the fluorescence recovery after photobleaching (FRAP) in the upper domain of the **MD5**. Panel II-Temporal normalized fluorescence intensity changes in the FRAP domain of the single **MD5**.

The fluidic properties of MDs **MD5** in the containment was demonstrated by the FRAP experiment, Figure S27. As shown in Figure S27(A), Panel I, the upper domain of a single MD was chosen as the bleach region of FRAP, where an intensified confined laser was applied for 2 s at t = 10 s, resulting in a totally FAM-bleached DNA constituents in the upper domain. Afterwards, the single MD was imaged at time

intervals to observe the fluorescence recovery process. At t = 230 s, the upper bleached domain is almost recovered, where the recovered fluorescence originated from the non-bleached DNA Y-shaped module **Y5** from the lower domain of the MD, demonstrating the fluid, dynamically exchangeable properties of the DNA Y-shaped module in **MD5**. Figure S27(A), Panel II showed the temporal normalized fluorescence intensities in the upper bleached domain of the single MD, the short recovery time of ca. 230 s indicated the fluid-like properties in the **MD5** containment, instead of gel-like properties.

Light-modulated transient formation/depletion of Mg^{2+}-ion-dependent DNAzyme-modified MD5

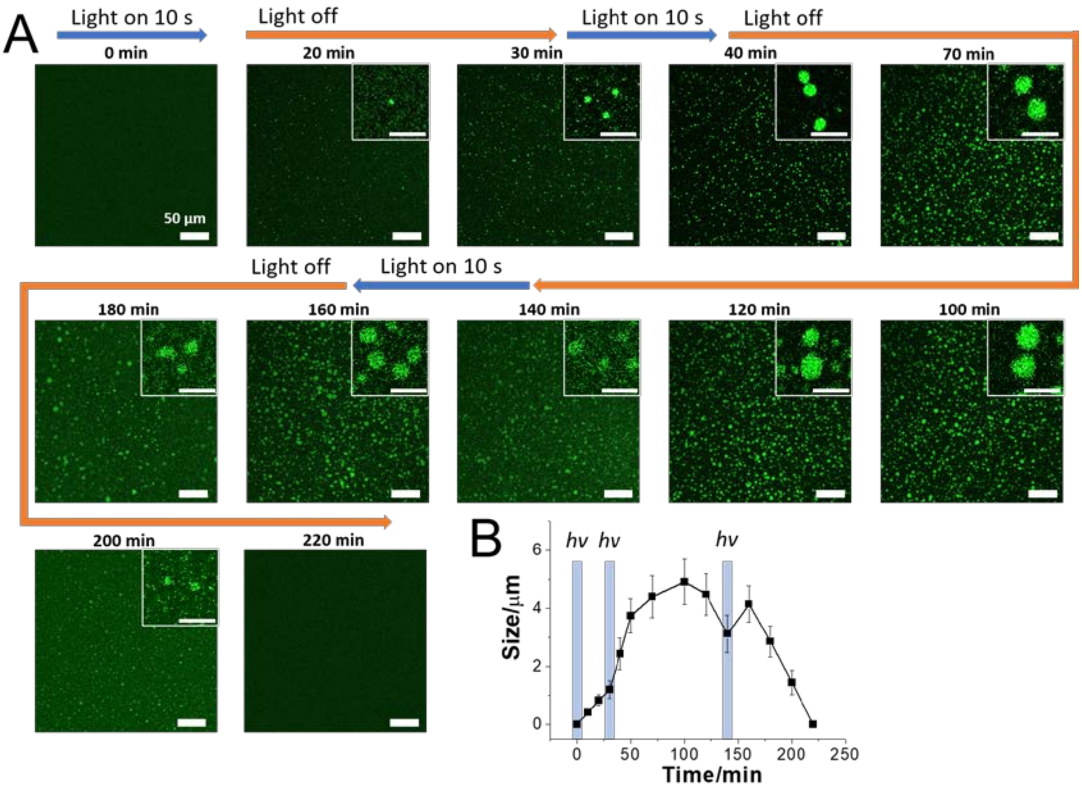

Figure S28. (A) Confocal fluorescence microscopy images (scale bar = 50 µm) corresponding to the light-triggered and light-modulated transient evolution/depletion of the MDs **MD5**. The primary light-triggered activation of the transient formation of the dissipative MDs **MD5** proceeds by applying a light pulse, (λ = 365 nm, P = 100 mW) for 10 s. At time t = 30 min and t = 140 min separately, a modulating light pulse, 10 s, was applied to refuel the dissipative system. (B) Temporal MDs size corresponding to the light-triggered and light-modulated evolution-depletion of the MDs. (Error bars in all experiments are derived from N = 3 experiments, analyzing in each experiment 4 imaged frames.)

As the photocaged hairpin could be partly uncaged by the dose of light irradiation, the amplitude of light-triggered transient assembly/depletion of Mg^{2+}-ion-dependent DNAzyme-functionalized **MD5** was supposed to be light-modulated by refueled light activation during the evolution and subsequent depletion process. Figure S28(A) and (B) displayed the temporal confocal fluorescence microscopy images and temporal

average size changes of **MD5** during the light-modulated transient evolution and depletion steps. The reaction module, composed of **Y5**, H_L, and H_M, was subjected to a short light illumination (λ = 365 nm, 10 s) activation, generating partial photo-uncaged H_L that could act as the fuel (**24**) to hybridize with **Y5** and generate self-crosslinked framework **MD5**. The evolution of phase-separated **MD5** proceeded at a slow rate, generating ca. 1.2 μm sized MDs within a time interval of 30 min. Then, the reaction module was re-fueled with a light pulse (λ = 365 nm, 10 s) activation. Re-fueling of the reaction module by more photo-uncaged H_L led to faster growth of the **MD5**, reaching ca. 4.9 μm at t = 100 min. Afterwards, the MDs was depleted because of the cleavage of metabolite hairpin H_M by DNAzyme units associated with the **MD5** and subsequent strand displacement of the fuel (**24**), leading to ca. 3.1 μm sized MDs at t = 140 min. The short light pulse could be applied further to modulate the sizes of the **MD5**, refueling the framework of **MD5** and generating larger MDs at t = 160 min. Afterwards, the framework of **MD5** was degraded slowly by the cleavage of metabolite H_M by DNAzyme units and eventually fully depleted within a time interval of 220 min.
